# Small ruminant production system characteristics and their influence on use, alternatives of, and resistance to antimicrobials in Nigeria

**DOI:** 10.1186/s12917-026-05577-7

**Published:** 2026-06-20

**Authors:** Mahmoud Eltholth, Rodiat Olabisi Omotoso, Ismail Adewuyi Adeyemo, Jadesola Juliana Ajao, Olayiwola Akeem Ahmed, Nma Bida Alhaji, Musa Imam Abubakar, Rodhiat Oyinlola Ade-Yusuf, Ibrahim Adisa Raufu, Victoria Olusola Adetunji, Jennifer Cole, Mabrouk Elsabagh, Elena Garcia Fruitós, Anna Aris, Ismail Ayoade Odetokun

**Affiliations:** 1 Department of Health Studies, Royal Holloway University of London, Egham, TW20 0EX UK; 2 Hygiene and Preventive Medicine Department, Faculty of Veterinary Medicine, Kafrelsheikh University, Kafr el-sheikh, Egypt; 3 Department of Statistics, University of Ilorin, Ilorin, 240003 Nigeria; 4 Department of Veterinary Microbiology, University of Ilorin, Ilorin, 240003 Nigeria; 5 African Centre of Excellence in Mycotoxin and Food Safety, Federal University of Technology, Minna, 930001 Nigeria; 6 Department of Theriogenology and Production, University of Ilorin, Ilorin, 240003 Nigeria; 7 Department of Veterinary Public Health and Preventive Medicine, University of Ilorin, Ilorin, 240003 Nigeria; 8 Department of Veterinary Public Health and Preventive Medicine, University of Ibadan, Ibadan, 200005 Nigeria; 9 College of Agriculture, University of Al Dhaid, Al-Dhaid, Sharjah, United Arab Emirates; 10 IRTA, Ruminant Production, Torre Marimon, 08140 Caldes de Montbui, Catalonia, Spain

**Keywords:** AMR, Sheep, Goats, Alternatives, Antibiotics, Herbs, Production, Nigeria, Ruminants

## Abstract

**Background:**

As the threat of antimicrobial resistance (AMR) escalates globally, the influence of indiscriminate antimicrobial use (AMU) in livestock cannot be overlooked. Antimicrobial use practices are continually explored in larger food-producing animals; however, small ruminants (sheep and goats) receive comparatively less research attention. Our study addresses this gap by investigating small ruminant production practices in Nigeria and exploring how they affect the use of antimicrobials and alternatives.

**Methods:**

We adopted a mixed-methods study design. A semi-structured questionnaire was administered to 785 farmers. Following this, a focus group discussion (FGD) was conducted with 23 small ruminant industry stakeholders, which included farmers, para-veterinarians, and butchers. Participants were split into three round tables, with 7–8 participants per table.

**Results:**

Of questionnaire respondents, 68% of farmers never vaccinated their flock against peste de petits ruminants (PPR) nor contagious caprine pleuropneumonia virus. Several health problems were regularly experienced by animals, including PPR, mastitis, and dermatophilosis. Diseases were mostly self-managed with antibiotics and herbs (> 70%) rather than through reliance on veterinary care (< 15%). More farmers (48%) used antibiotics than herbal remedies (14%) over the previous three months. Farmers’ use of herbs was affected by their having low awareness of available options and how to use them appropriately. Perceived effectiveness also influenced farmers’ choice between antibiotics and herbs, while economic considerations also led them to sell off sick animals before or during treatment. Among farmers who used animal health services, more farmers (59%) consulted unlicensed para-veterinarians and drugstore vendors rather than licensed government and private veterinarians (36%), a disparity attributed to the unavailability of qualified veterinary doctors. Most farmers had poor knowledge (62%), attitudes (47%), and practices (43%) towards AMU and AMR.

**Conclusions:**

We recommend conducting further studies to identify and investigate the efficacy of currently used herbs in treating common diseases. There is a crucial need to improve farmers’ access to vaccines, veterinary care, and laboratory diagnostics. Barriers that hinder better compliance with regulations that govern the use of non-prescribed antimicrobials should be explored. Awareness programmes could be conducted to improve farmers’ awareness of AMR and appropriate disease preventive practices.

**Graphical abstract:**

**Figure:**
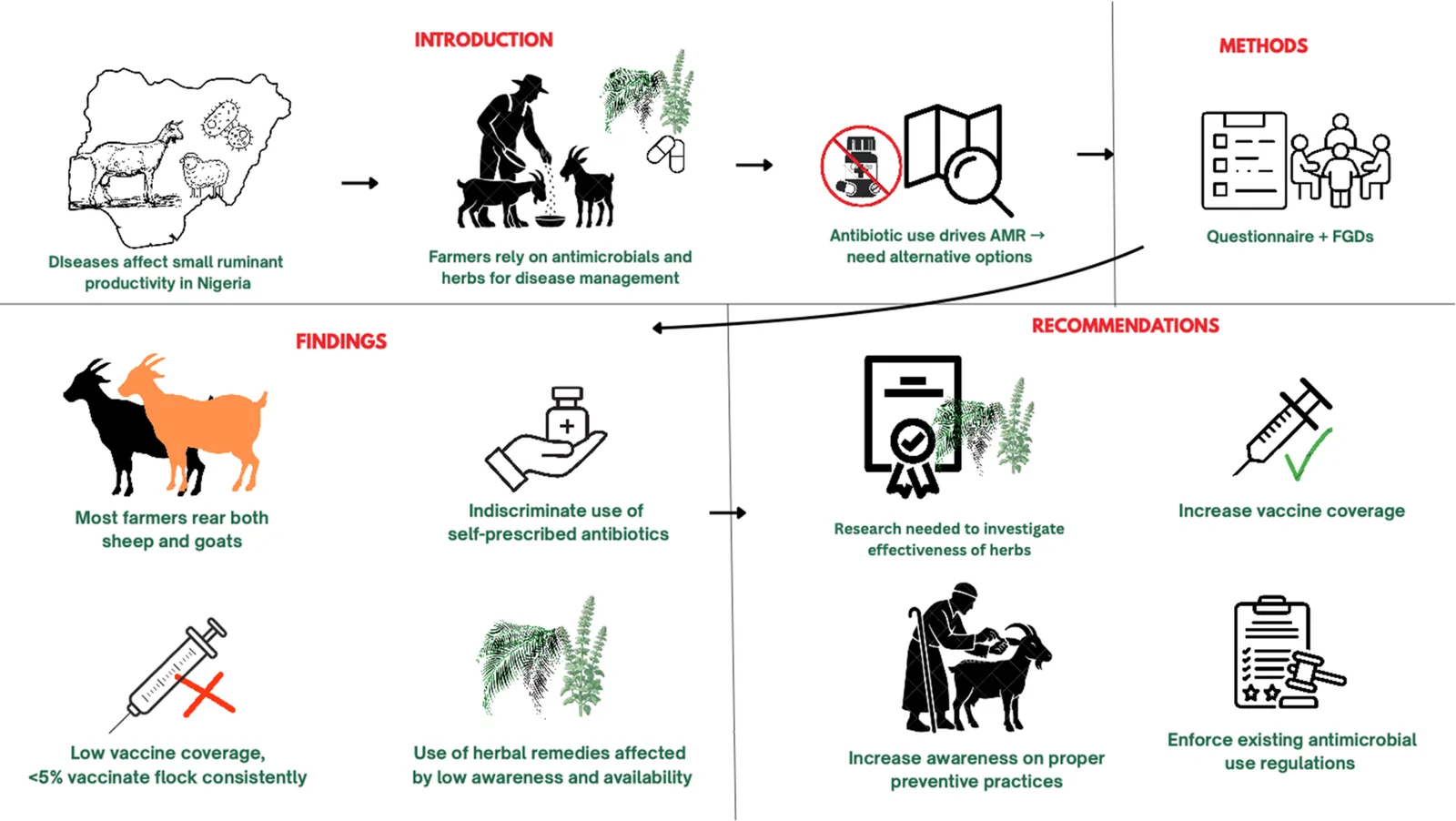

**Supplementary Information:**

The online version contains supplementary material available at https://doi.org/10.1186/s12917-026-05577-7.

## Highlights

- 48% of farmers self-administered antibiotics within the last three months.

- Less than 5% of farmers vaccinate their flock regularly.

- 58% of farmers do not use antimicrobial alternatives in their production.

- Low awareness and availability of herbal remedies affect farmers’ use.

- Economic considerations cause farmers to sell off ill and dying animals before or during treatment.

**Supplementary Information:**

The online version contains supplementary material available at https://doi.org/10.1186/s12917-026-05577-7.

## Background

Livestock production plays a crucial role in Nigeria’s agricultural sector, accounting for roughly one-third of the country’s agricultural gross domestic product [1]. Small ruminants are a substantial portion of Nigeria’s livestock, with an estimated population of 19 million sheep and 28 million goats [2]. Small ruminant production is particularly prominent in rural communities, as it contributes substantially to food security and maintaining incomes in low- and middle-income households [3, 4]. In many cases, households integrate small ruminant farming with cattle and/or crop production to maximise general output and reduce costs [5, 6]. Most farmers adopt extensive and semi-intensive grazing systems, in which animals rely largely on outdoor forages and refuse dumps in the environment. These systems increase animals’ risk of exposure to diseases endemic in the environment [7, 8].

Diseases commonly affecting small ruminants in Nigeria include mastitis, peste des petits ruminants (PPR), and poxviruses [9–11]. A study on PPR in Nigeria estimated its prevalence to be 6% in sheep and 21% in goats [9]. Another study estimated the incidence of mastitis as 40.4% across three states in Nigeria, 30.4% of which were subclinical mastitis cases with no obvious physical signs [11]. These diseases are considered to be widespread and circulating actively across Nigeria, leading to increased morbidity and mortality within the affected flock [9–11]. In this regard, mastitis is a particularly significant disease, as it causes consistent underperformance in terms of milk production and increases kid mortality due to resultant starvation [11].

To curtail these losses, farmers rely on antimicrobials, especially antibiotics, for health management [12, 13]. These drugs are often misused. A study on pastoralist ruminant farmers in Nigeria observed that 65% of farmers did not consult veterinary services and rather practiced self-administration. 67% of farmers administered antibiotics arbitrarily and without clear protocols, and only 16% observed withdrawal periods after antibiotic use (ABU) [12]. Indiscriminate antimicrobial use (AMU) in this manner has led to the deposition of sub-therapeutic dosages of antimicrobial residues in the gut of animals, conferring selection pressure and leading to the development of antimicrobial-resistant genes (ARGs) in infectious microbes [12–14]. This pattern has also been observed across other countries in the world, for instance, in a Portuguese study, 83% of samples obtained from 65 small ruminant farms contained at least one ARG [14]. Through non-observance of withdrawal periods in food-producing animals, ARGs can be passed from livestock to humans and the environment through risk pathways of milk consumption and poor manure disposal [14]. Antimicrobial residues have also been implicated in human allergic reactions, damage to vital organs such as the kidneys, liver, and bone marrow, increased cancer risk, and developmental problems in foetuses and children [14, 15]. The most undesirable effect of ARGs, however, is the replication of AMR, a significant global health threat projected to cause 10 million human deaths annually by 2050 [15, 16].

Currently, efforts are being put in place globally to reduce AMU in livestock production through the development and use of antimicrobial alternatives (ATAs). ATAs are tools and substances that can prevent or control infections, thereby reducing the need for antimicrobials and subsequently the risk of AMR. These include vaccines, herbal plants, antimicrobial peptides, bacteriophages, and probiotics [17]. Unfortunately, several ATAs have significant hindrances that prevent them from being functional and effective replacements for antimicrobials. For instance, vaccines are available in Nigeria for poxviruses and PPR; however, low vaccination coverage and a general lack of awareness reduce the potential for appreciable impact in this regard [18, 19]. There is little evidence that vaccines widely used elsewhere are utilised for small ruminant mastitis in Nigeria, due to relatively low research and development efforts in these areas. In addition, studies from other countries report that currently available mastitis vaccines generally have low to inconsistent levels of efficacy, with protection declining over time [20]. Improved hygiene practices are regarded as more reliable ATAs for preventing mastitis [20]. However, hygienic management for mastitis prevention is inconsistently utilised by Nigerian ruminant farmers [20, 21]. Another group of ATAs used in Nigeria is herbal plants. Herbs such as *Solanum spp* and *Glyphea brevis* have been used in Eastern Nigeria for diarrhoea and retained placenta in small ruminants. These herbs have been recorded to have antimicrobial properties, affirming their effectiveness for these conditions [22]. Farmers also utilise regular household materials such as kerosene, palm oil, and salt for treating infectious diseases [22, 23]. However, some studies have noted that a deeper understanding of the mechanism of action of these herbs, as well as their effective dosage concentrations, is still lacking, preventing their full adoption by Nigerian farmers [17, 22, 23].

There exist clear evidence gaps regarding the current use of ATAs in Nigeria, as well as an understanding of the factors that restrict small ruminant farmers from using them when compared to antimicrobials. Thus, this study presents an exploratory sequential mixed-methods approach aimed at characterising the small ruminant production system in North-central Nigeria and identifying possible influences on the use of antimicrobials and ATAs in the management of infectious disease conditions.

## Materials and methods

### Study area

**Fig. 1:**
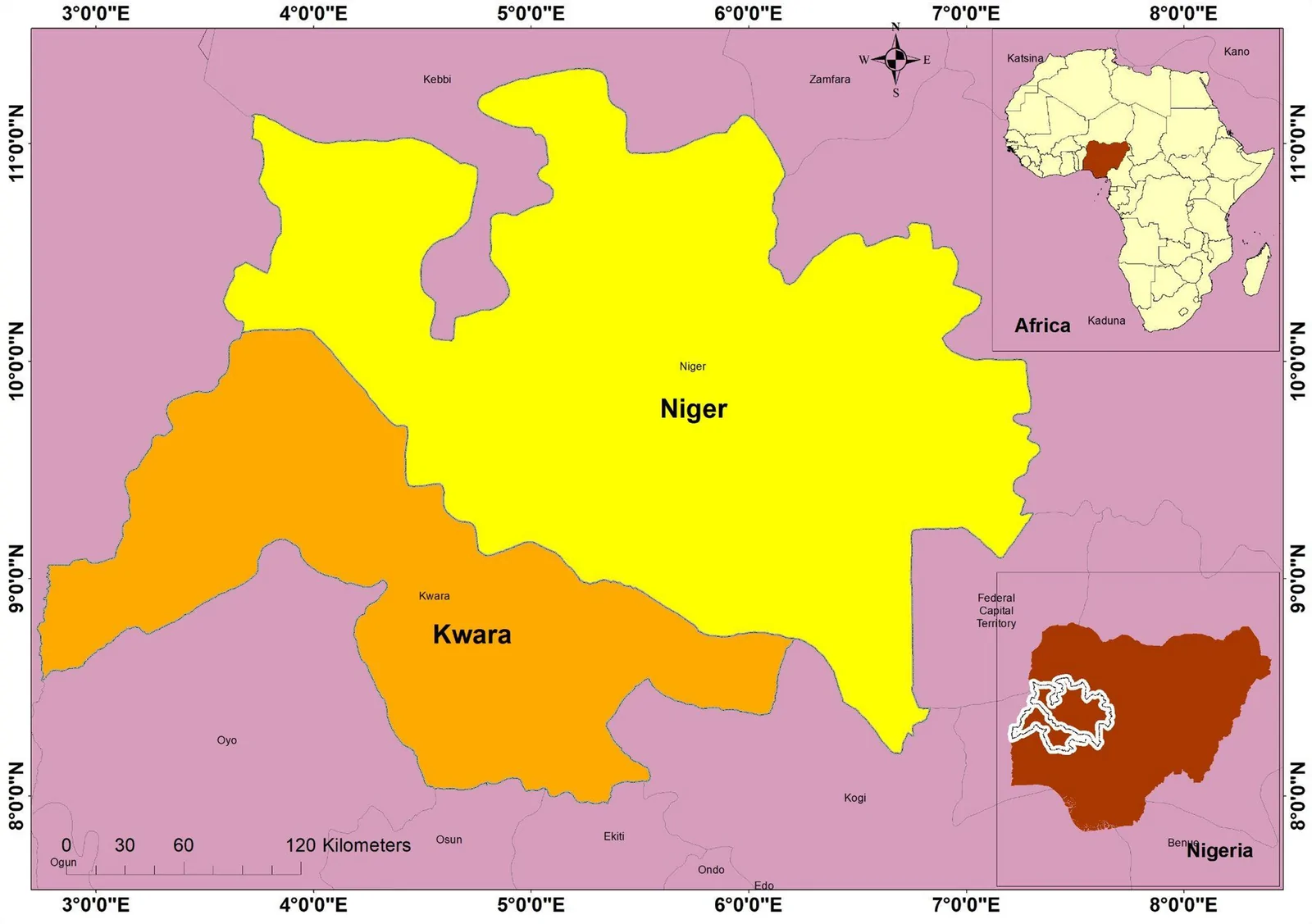
Geographical locations (Niger and Kwara states, Nigeria) of the study participants

The study was conducted in two predominantly ruminant-farming states in North-central Nigeria: Kwara and Niger states (Fig. 1). Kwara State is located between latitude 8° 05’ N and 10° 05’ and longitude 2° 50’ E to 6° 05’ E, while Niger State lies approximately between 8° 00′ N and 11° 30′ N latitude and 3° 30′ E to 7° 40′ E longitude. Both states are located in the Guinea savanna zone of Nigeria, characterized by mixed crop-livestock production systems run by smallholder farms. Livestock rearing is mostly pastoral. The region experiences a tropical climate with mean annual temperatures between 25 and 37 °C and annual rainfall from 1,000 to 1,500 mm [24, 25]. In Niger State, there are approximately 1 million sheep and 2.6 million goats [24]. Niger State and Kwara State are divided into 3 and 4 Agricultural Development Zones (ADPs), respectively [24, 25].

### Study populations

The target populations for the questionnaire survey included small ruminant farmers, herders, and farm workers within the study area. Small ruminant farmers outside Kwara and Niger states, or livestock farmers who did not keep small ruminants in their herds, were excluded.

### Study design

We adopted a mixed-methods study design comprising a cross-sectional questionnaire survey and a focus group discussion of key stakeholders in the small ruminant farming industry in the study area. The study was conducted in four phases from January 2025 to February 2026. Phase I consisted of the design and administration of the questionnaire survey (January to March 2025). In Phase II, survey data were compiled and analysed (March to September 2025). Phase III involved reviewing the quantitative results and organising a focus group discussion (FGD) to generate further insights **(**October 2025). Phase IV involved qualitative thematic analysis and report writing (October 2025 to February 2026).

### Sample size determination and sampling techniques

**Fig. 2:**
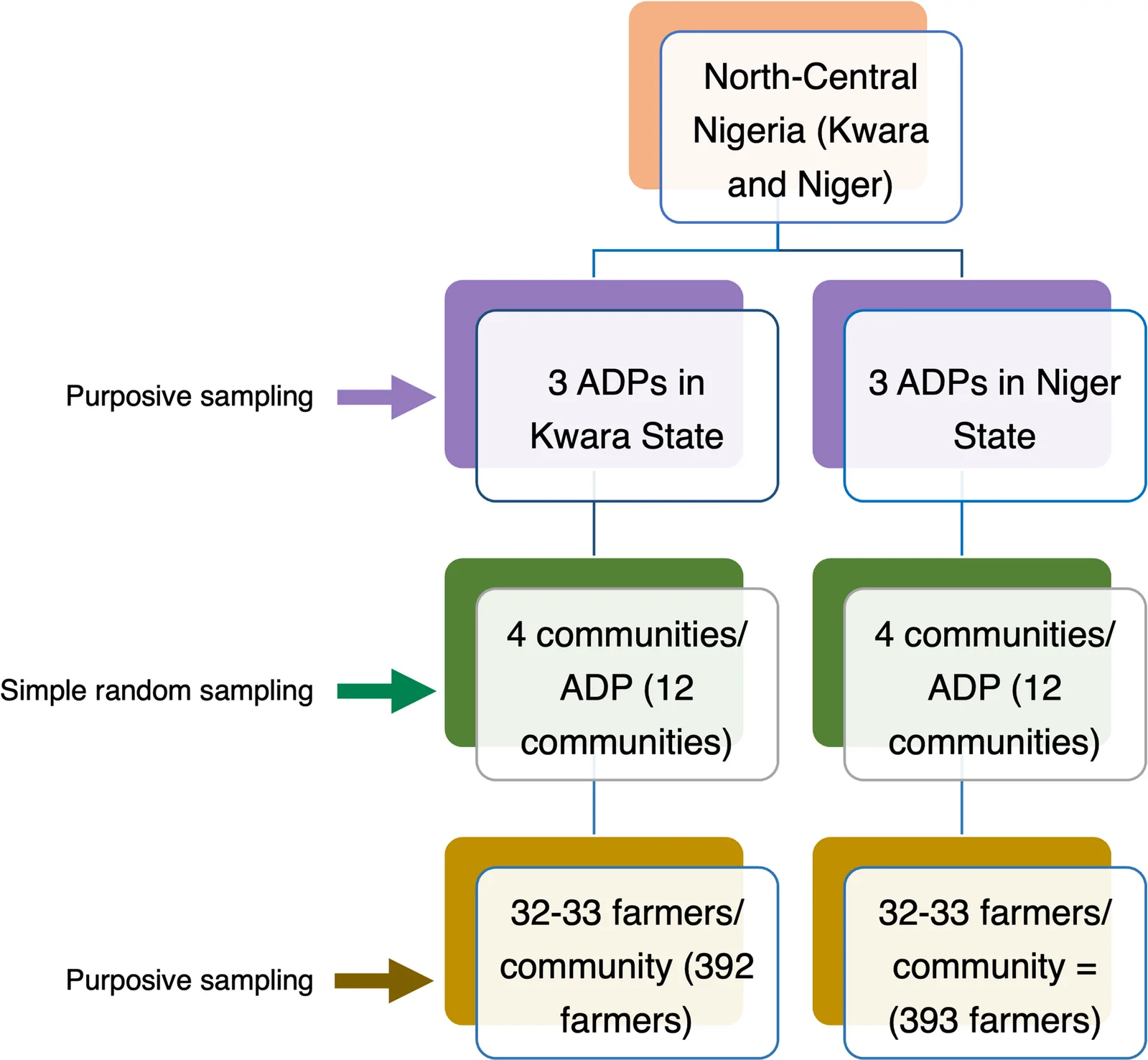
Multistage sampling technique used to recruit respondents for the questionnaire survey

Multistage sampling techniques were employed to select respondents (Fig. 2). The first stage involved the selection of three ADPs in each state. For Kwara State, the three zones with the highest ruminant farming activities were purposively selected. The second stage involved a random selection of 4 pastoral communities from each zone (12 communities per state). Communities selected in Kwara State were Amoyo, Lajiki, Amberi/Ijagbo, Ajase-Ipo, Kaiama, Ilesha Baruba, Okuta, Kageji, Fufu, Afon, Oke Oyi, and Tafatafa. In Niger State, selected communities included Bida, Agaie, Bosso, Edati, Gbako, Gurara, Katcha, Lapai, Lavum, Mokwa, Agwara, and Suleija. The third stage involved a purposive sampling of 32–33 appropriate respondents from each community. The sample size was determined to be 384 per state via OpenEpi, with an estimated proportion of 50%, a 5% margin of error, and a 95% confidence level. Ultimately, 785 respondents were surveyed to account for possible incomplete or non-responses.

For the FGD, 23 stakeholders were recruited from the Nigerian small ruminant farming industry in Kwara and Niger states, including 17 small ruminant farmers who had earlier filled the questionnaire, 3 para-veterinarians, and 3 butchers/meat vendors.

### Designs of questionnaire and interview guide

The research team developed the questionnaire following a series of meetings and a literature review of similar studies conducted in Nigeria [26, 27]. The questionnaire was developed in English and administered by researchers who were at least fluent in two of four languages: English, Fulani, Hausa and Yoruba. The researchers attended two training sessions to enhance their capacity in translating and administering the questionnaire. The questionnaire was piloted with 10 farmers in a pastoral community to ascertain its validity, after which necessary revisions were made. The pilot survey was conducted separately, and the findings were not included alongside the main survey. The final version consisted of five sections: (a) farmers’ socio-demographics, (b) farm characteristics, (c) livestock management practices, (d) animal health management practices, and (e) knowledge, attitudes, and practices (KAP) of AMU and AMR (Supplementary File 1). The questionnaire was semi-structured and comprised single-choice, multiple-choice, and open-ended questions.

Following the questionnaire survey, the research team reviewed the findings and highlighted key areas that required further exploration. Subsequently, a focus group discussion was organised. An interview guide was developed via team meetings. The guide comprised 11 questions focused on farmers’ health management practices, the economic impact of diseases, and factors that affected the use of antibiotics, ATAs, and animal health service providers **(** Supplementary File 2).

### Data collection

The questionnaire survey was administered through face-to-face interviews. Received responses were collated in English on paper or electronic forms via KoboToolbox (v.2.024.36). Paper forms were checked for completeness before inputting into KoboToolbox. The link is attached here: https://ee.kobotoolbox.org/x/N6rwhJOq. During the questionnaire survey, antimicrobials were defined to farmers as all anti-infectious medications used in treating infectious diseases, including antibiotics, antifungals, and anthelminthics.

During the FGD, participants were split into three round tables, with 7–8 participants per table. For each table, a trained moderator was assigned to lead the discussion using the interview guide. A co-moderator/note taker was taking notes and, when possible, recording the conversation electronically for each table.

### Statistical analysis

Questionnaire data were exported to Microsoft Excel and cleaned. The cleaned data were then imported into R (v. 4.5.1). Findings were summarized descriptively using frequencies, proportions, averages, and ranges. For the KAP, the farmers were asked 4 knowledge questions, 10 attitude questions, and 12 practice questions. Under the section, knowledge questions were scored 1 for a correct answer and 0 for ‘Yes’ and ‘No,’ respectively. Attitude questions were scored on a 3-point Likert scale (0 = Disagree/ I don’t know, 1 = Neutral, 2 = Agree). Practice questions were answered ‘Most likely’, ‘Likely’ or ‘Not Likely’, which were given scores of 2, 1 and 0, respectively. Negative questions in each section were scored in reverse. To enable relative comparison within our study population, we obtained a data-driven cut-off using the group mean, consistent with previous studies [28, 29]. The above-mean section included scores above the population average (≥ 1.23 for knowledge, ≥ 8.46 for attitudes, and ≥ 11.43 for practices). As questions were all scored with whole numbers, this translated to ≥ 2 for knowledge, ≥ 9 for attitudes, and ≥ 12 for practices. Pearson’s chi-square test (bivariate analysis) was used to further test the associations between KAP scores and farmers’ demographic characteristics. All significant associations at the *p* < 0.25 level in the bivariate analysis were subsequently entered into a multivariate logistic regression model to test for independent associations. Confounding was accounted for through the multivariate logistic regression. Finally, a Spearman’s correlation test was conducted to determine the associations between the three scored groups.

Audio recordings were transcribed manually and automatically using the TurboScribe software. Supplementary notes taken by the co-moderator captured nonverbal cues and group dynamics. Thereafter, analysis was conducted based on Braun and Clarke’s guide to reflexive thematic analysis [30]. The analysis was deductive with a majorly semantic coding approach. First, transcripts were read multiple times to allow for data familiarisation. Visual maps were also created to aid researchers with retention. Two independent researchers generated initial codes. Afterwards, researchers came together to search for potential themes. Final themes were decided after multiple rounds of discussion and reflection. These themes have been explored in the Results section. Thematic interactions were integrated into a rich picture using Lucidchart to illustrate interactions between factors influencing farmers’ health management decisions.

## Results

### Demographic characteristics

**Fig. 3:**
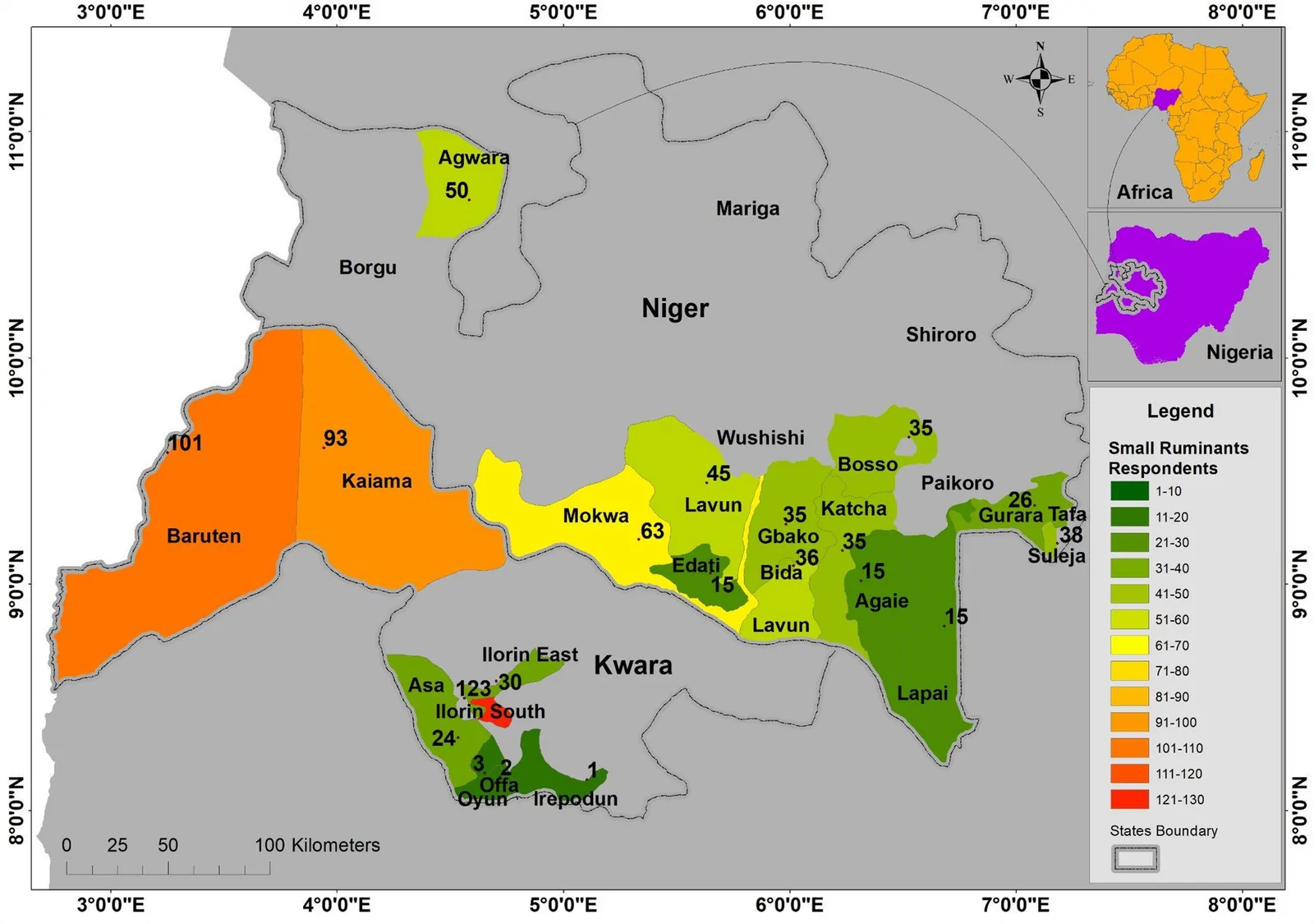
Geographical distribution of study respondents across Kwara and Niger states, Nigeria

Demographic characteristics of 785 survey respondents are summarised in Table 1, with geographical distribution in Fig. 3. Most respondents (60.8%) were between 31 and 50 years of age. The majority of respondents (78.1%) were male. Most (65.7%) had more than 10 years of farming experience. The majority (82.2%) owned fewer than 50 small ruminants in their flock.

**Table 1:** Demographic characteristics of farmers

Demographics	*n* (%)
Age (years)	
≤ 20	8 (1.0)
21–30	130 (16.6)
31–40	277 (35.3)
41–50	200 (25.5)
51–60	132 (16.8)
> 60	38 (4.8)
Gender	
Male	613 (78.1)
Female	172 (21.9)
State	
Kwara	377 (48.0)
Niger	408 (51.9)
Level of Education	
No formal education	461 (58.7)
Primary School	114 (14.5)
Secondary School	128 (16.3)
Tertiary Education	82 (10.5)
Role on Farm	
Owner	461 (58.7)
Owner and manager	248 (31.6)
Manager	51 (6.5)
Worker	25 (3.2)
Number of years in ruminant farming	
≤ 10	269 (34.3)
11–20	258 (32.9)
21–30	145 (18.5)
31–40	74 (9.4)
> 40	39 (4.9)
Herd size of animals	
≤ 25	299 (38.1)
26–50	346 (44.1)
51–75	81 (10.3)
> 75	59 (7.5)

### Farm characteristics and management practices

Most farms (65%) kept a mixed herd of sheep and goats. The largest herd segment was females above prime reproductive age for both species (> 2 years) (Supplementary Table 1). Fewer than 40% of farmers routinely kept feeding, reproduction, or health records (Table 2). Flocks (54.2%) were mostly housed in an open area without netting, and a significant proportion (46.8%) was reared in an extensive grazing system. The majority of farmers (83.9%) relied on natural pasture to feed their flock (Supplementary Fig. 1).

**Table 2:** Farm characteristics and management

Farm Characteristics and Management	*n* (%)
Housing systems	
Open housing system without netting	425 (54.1)
Open housing system with netting	221 (28.2)
Closed housing system	139 (17.7)
Feeding practices	
Extensive system	367 (46.8)
Semi-intensive system	306 (38.9)
Intensive system	112 (14.3)
Purchase replacement animals?	
Yes	364 (46.4)
No	421 (53.6)
Use of community grazing lands	
Yes	476 (61.7)
No	272 (35.3)
I don’t know	23 (2.9)
Record keeping	
Feeding	214 (27.3)
Reproduction	263 (33.5)
Veterinary Health Products	244 (31.1)
Vaccination programs	179 (22.8)

### Prevalent diseases and farmers’ health management practices

The most prevalent diseases affecting the small ruminant flock were mastitis, dermatophilosis, and PPR. Some farmers (13.9%) had experienced mastitis in their flock over the last 12 months. FGD participants who also reared cattle stated the disease was more prevalent in small ruminants than in cattle: *“We can go 10 years at a time without seeing [mastitis] in our cattle*,* but we usually experience it in [our] sheep and goats.” – R3*,* small ruminant farmer.* Of the farmers surveyed, 69.8% had no idea of the origin of mastitis, while 18.3% believed the disease originated from poor management. Farmers regularly put up their animals for human consumption after mastitis treatment failure, as in this account: *“I have experienced [mastitis] and if it doesn’t [improve after treatment]*,* we just sell it [to the butchers].” – R07*,* small ruminant farmer.* Dermatophilosis was likewise considered a significant burden due to its regular occurrence and the loss incurred by farmers once it was discovered: “*In 10 animals*,* we [usually] have 1–2 cases [of dermatophilosis]. From experience*,* if we see dermatophilosis on our farm*,* we sell them out immediately at a loss of 75%.” - R12*,* small ruminant farmer.* PPR was considered one of the most severe diseases, as it led to substantial mortalities: “*My major challenge is [PPR]. I had small ruminants but [PPR] wiped out the whole stock.” - R01*,* small ruminant farmer.* Other, less prevalent infections included foot rot and lameness.

Most farmers surveyed (68.2%) did not vaccinate their flock against PPR and Contagious caprine pleuropneumonia (CCPP). Within the last year, 28.4% of farmers had their flocks vaccinated against PPR and 3.3% against CCPP (Table 3).

**Table 3:** Animal health management practices of farmers

Animal Health Management Practices	*n* (%)
Vaccination	
Never	535 (68.2)
Sometimes	214 (27.3)
Always	36 (4.6)
Use of antimicrobial alternatives (ATAs) in animal production	
Yes	141 (17.9)
No	455 (57.9)
I don’t know	189 (24.1)
Diseases vaccinated against in the last year	
PPR	223 (28.4)
CCPP	26 (3.3)
Anthrax	21 (2.7)
Blackquarter	3 (0.4)
Use of animal health services over the last 3 months	
Yes	383 (48.8)
No	397 (50.6)
I’m not sure	5 (0.6)
Actions taken to protect against mastitis	
Do nothing	589 (75.0)
Proper management	190 (24.2)
Conventional treatment	4 (0.5)
Alternatives to antibiotics	2 (0.3)
Methods used to currently detect mastitis	
Observation	513 (65.4)
Laboratory tests	16 (6.0)
I don’t know	256 (32.6)
First step on suspicion of mastitis	
Do nothing	405 (51.6)
Consult a veterinarian or para-veterinarian	235 (29.9)
Use self-prescribed antibiotics	85 (10.8)
Use ATAs	40 (5.1)
Isolation of the affected animal	14 (1.8)
Culling and selling	6 (0.8)

**Fig. 4:**
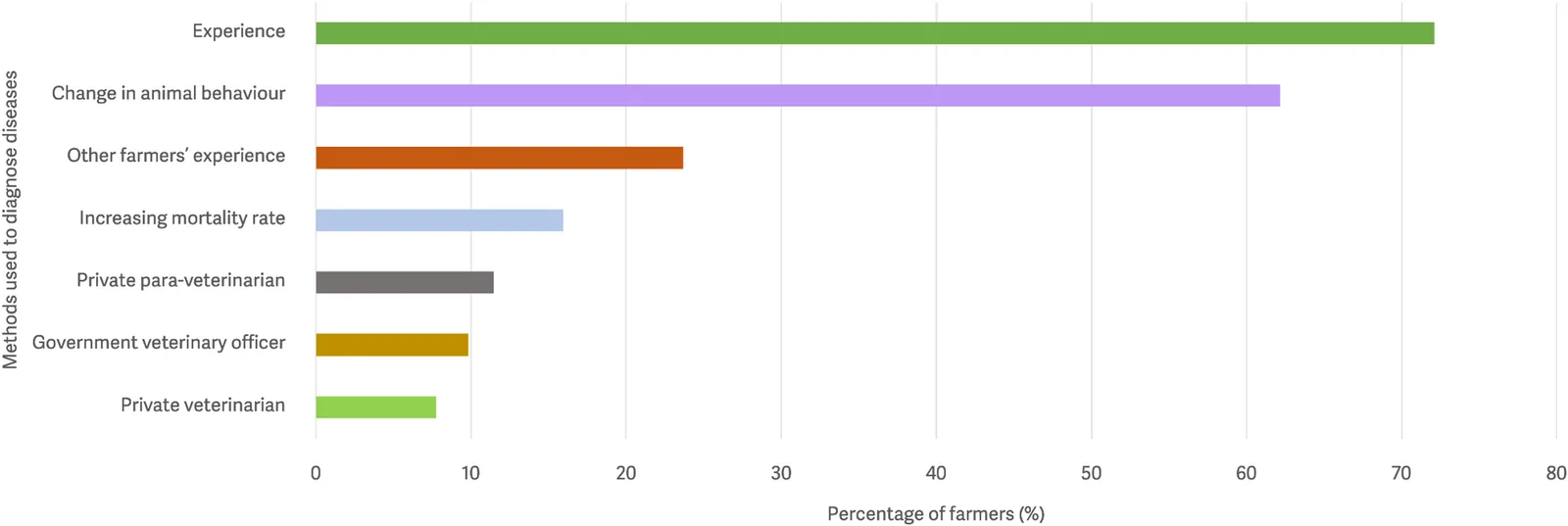
Methods used by farmers in diagnosing diseases

**Fig. 5:**
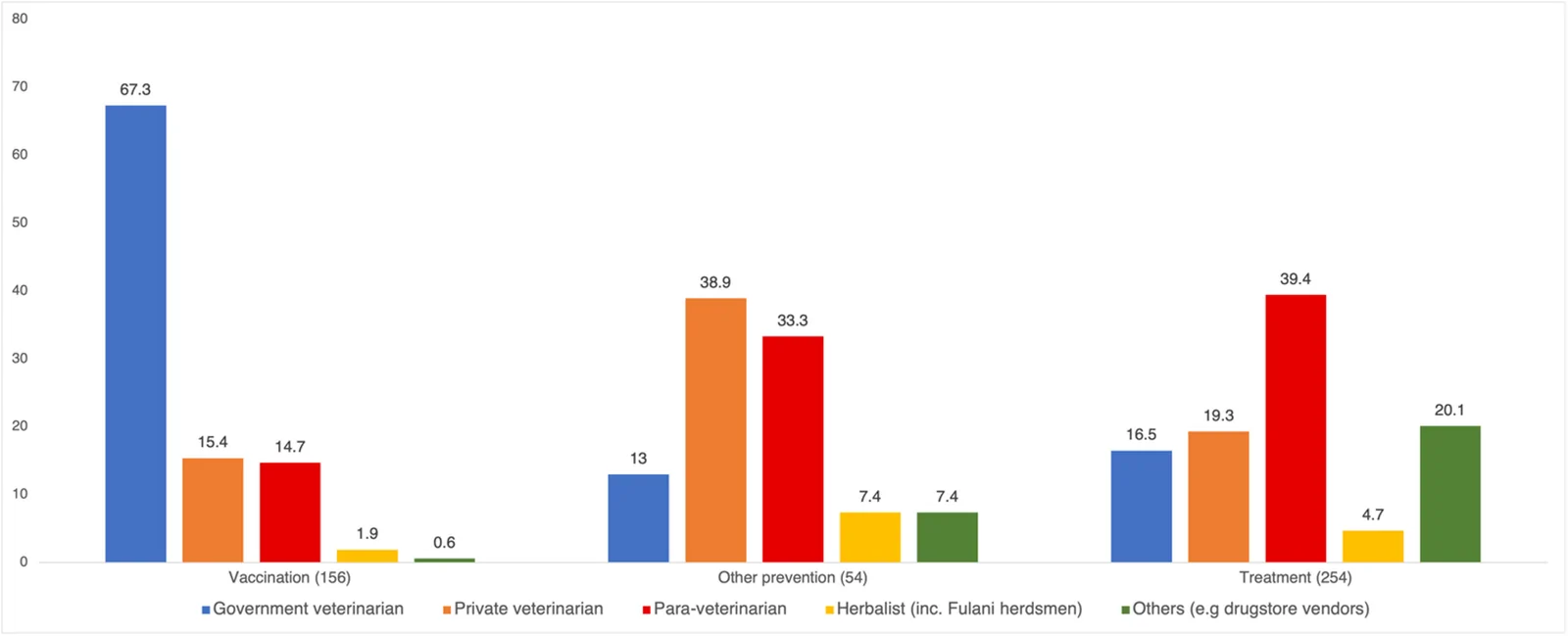
Animal health service providers used over the last three months, according to the services provided

**Fig. 6:**
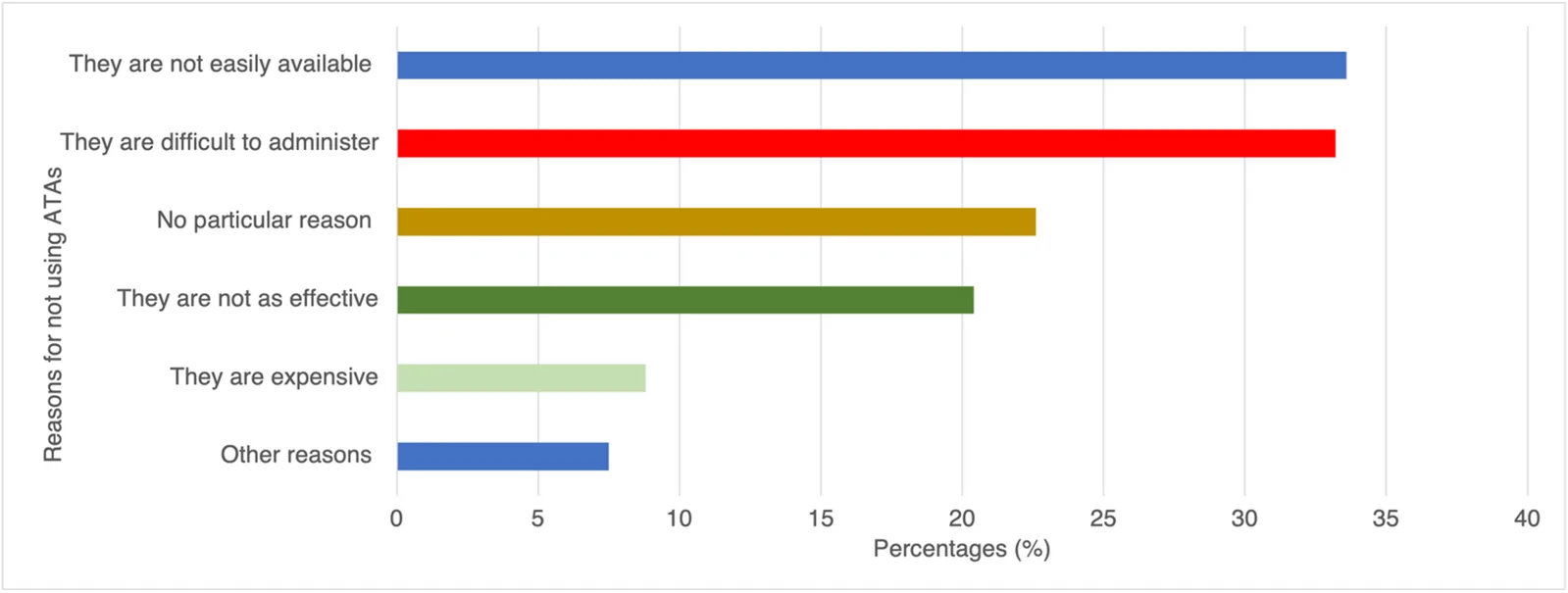
Reasons farmers (*n* = 455) provided for not using antimicrobial alternatives

In diagnosing diseases experienced, farmers primarily relied on their experience (72.1%) and changes in animal behaviour (62.1%), with far less focus on veterinary consultation (< 15%) (Fig. 4). Over the last three months, 383 (48.8%) of farmers had consulted various animal health services. Figure 5 summarises the animal health services used and services provided. Of the 156 farmers who had vaccinated their animals against PPR or CCPP within the time period, 67.1% relied on government veterinarians. For treatment, farmers mostly consulted para-veterinarians (39.4%) and drugstore vendors (20.1%) rather than government veterinarians (19.3%) and private veterinarians (farmers (16.5%). Para-veterinarians are people relied on by farmers to treat diseases primarily through the use of antimicrobials, but who did not possess valid veterinary licenses. Government veterinarians are licensed professionals paid by the government to dispense veterinary services in the community, with their services ranging from free to cheaply affordable. Private veterinarians had to be directly hired by farmers, and their services were more expensive. Herbalists used local herbs in treating diseases, and this group largely included Fulani herdsmen, traditional pastoralists familiar with ethnoveterinary remedies. More than half (58.0%) of farmers did not use ATAs in their ruminant production. Reasons for this included unavailability (33.6%) and their being difficult to administer (33.2%) (Fig. 6). There were no statistically significant associations between the use of ATAs and farmers’ demographics (*p* > 0.005).

**Fig. 7:**
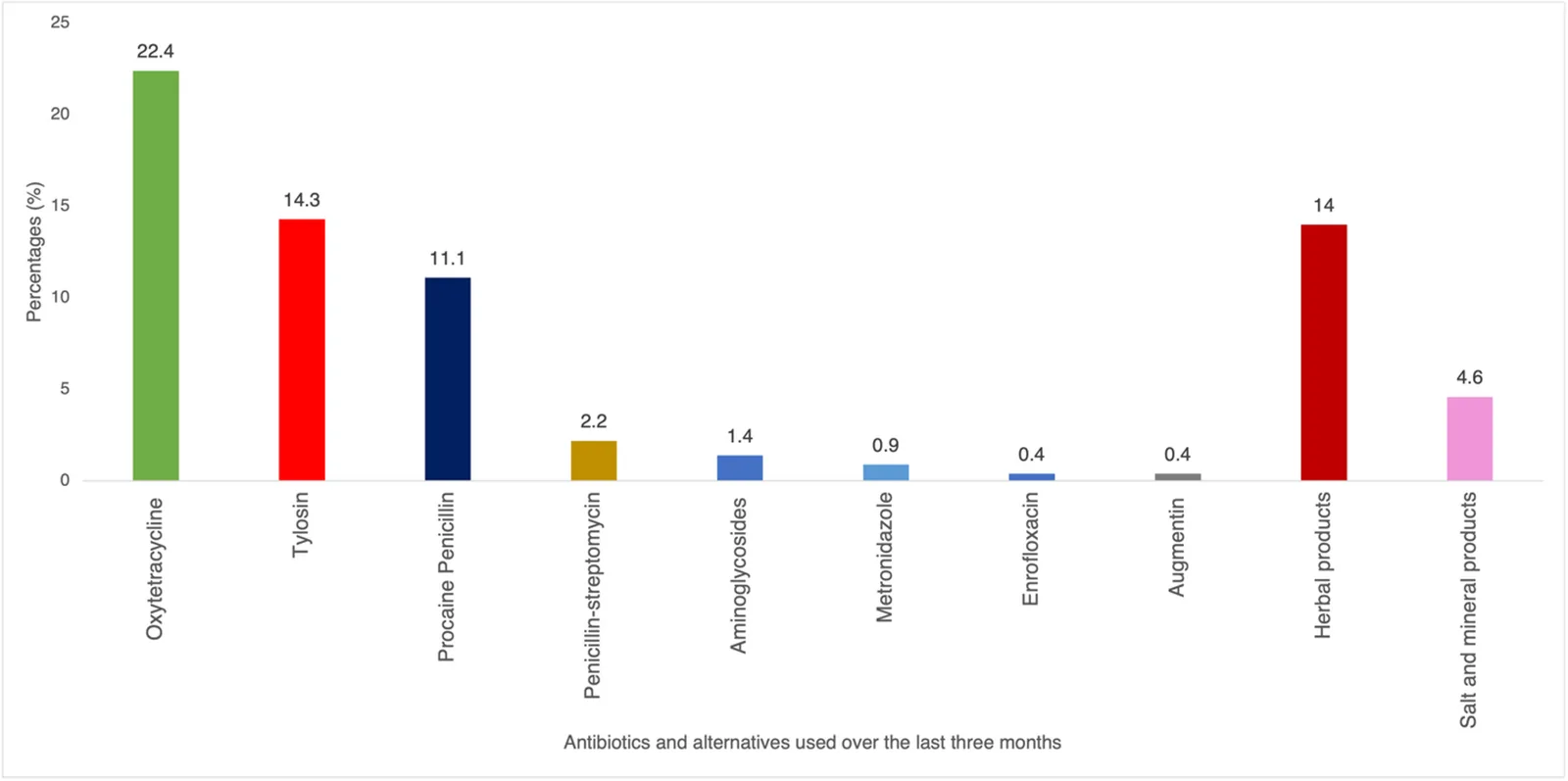
Antibiotics and ATAs used by farmers over the last three months

A significant proportion of farmers (47.6%) had self-prescribed antibiotics to their herd over the last three months. The most commonly prescribed antibiotics included oxytetracycline (22.4%), tylosin (14.3%), and procaine penicillin (11.1%) (Fig. 7).

These antibiotics were primarily sourced from (unlicensed) animal feed stores, veterinarians, and livestock markets (Supplementary Fig. 2). Over the last three months, 14.0% and 4.6% of farmers reported using herbal plants and salt as ATAs, respectively. Herbs included *Dongoyaro* (Yorubá and Hausa name for neem plant or *Azadirachta indica*), *Àgánwò/Kahi (*Yorubá and Fulani names for mahogany hardwood or *Khaya senegalensis)*, bitter leaf plant (*Vernonia amygdalina)*,* Kadanya* (Fulani name for shea butter tree or *Vitellaria paradoxa*),*ígí ìpín* (Yorubá name sandpaper tree or *Ficus exasperata*). FGD participants stated that the neem plant was used for a variety of diseases. Mahogany hardwood was used in treating mastitis and CCPP, while sandpaper tree was used for mastitis. Farmers also used some materials such as potash, shea butter, heat, lime, and beans for various diseases. One farmer described using beans to treat mastitis as follows: *“I don’t use conventional drugs. I use the herbal way*,* with bean peel. For the [animals] whose teats are hard and there is no milk*,* we give [beans peel] to them. […] We also feed it [to the animals] and [the teat] will become soft.” - R09*,* small ruminant farmer.*

### Knowledge, Attitudes and Practices (KAP) towards antimicrobials and AMR

On average, farmers demonstrated poor knowledge and attitudes about antibiotics, but showed higher scores for practices (Table 4). Among the knowledge findings, 55.5% and 31.9% of farmers had heard of the terms ‘antibiotic’ and ‘AMR’ respectively. Only 14.8% of farmers knew that mastitis could be caused by AMR pathogens.

**Table 4:** Total KAP scores for farmers

KAP (Total possible score)	Mean score	Above mean *n* (%)	Below mean *n* (%)
Knowledge (4)	1.2	299 (38.1)	486 (61.9)
Attitudes (20)	8.5	418 (53.3)	367 (46.8)
Practices (24)	11.4	447 (56.9)	338 (43.1)

**Fig. 8:**
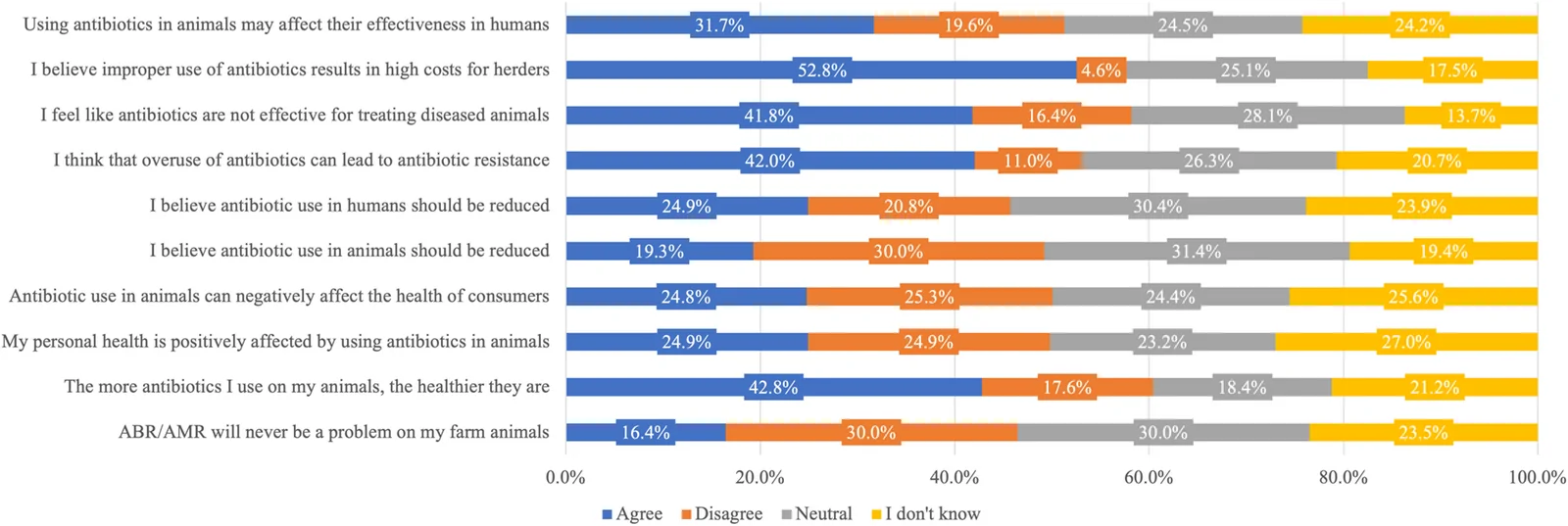
Farmers’ attitudes regarding the use of antibiotics

**Fig. 9:**
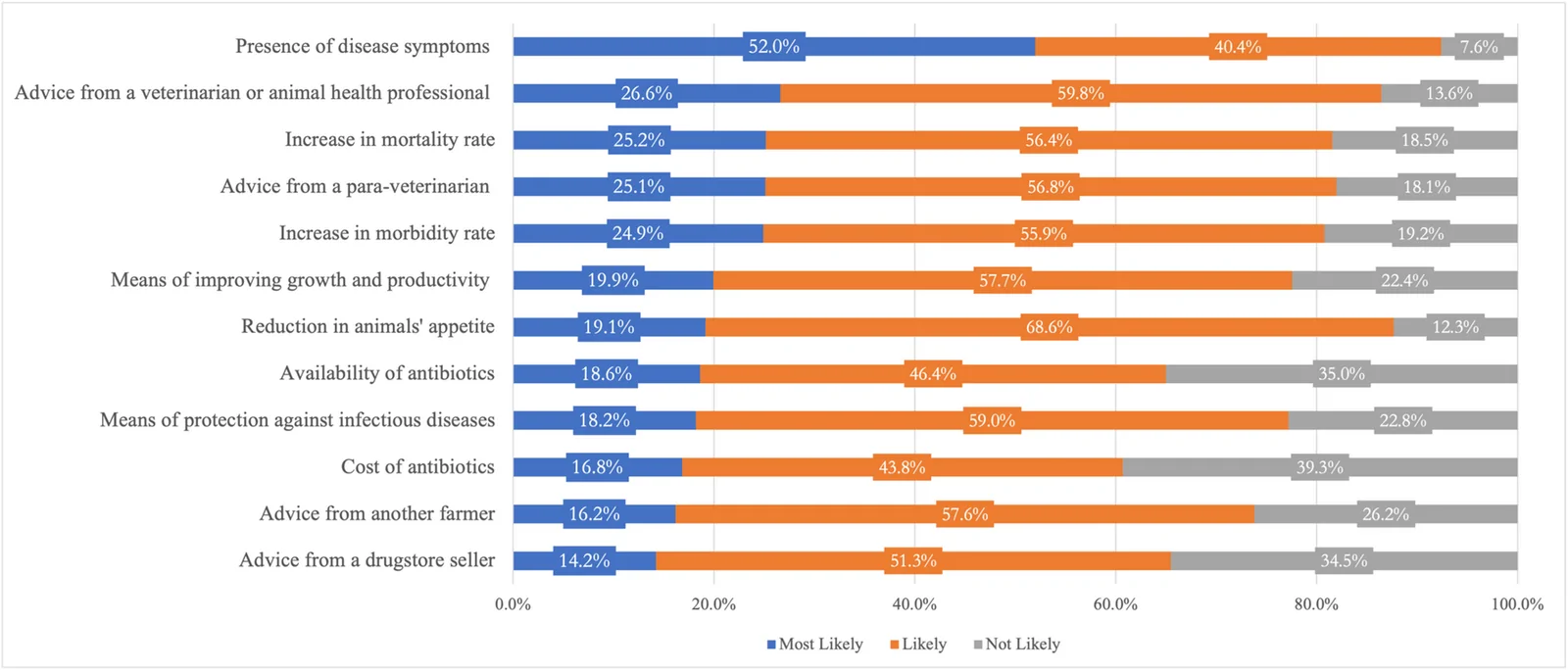
Factors influencing farmers’ antibiotic practices in their herd

Farmers’ attitudes regarding AMU and AMR are summarized in Fig. 8. Less than half of the farmers surveyed (42.0%) agreed that overuse of antibiotics could lead to resistance, and 41.8% of farmers agreed that antibiotics were no longer effective. Less than one-third (30.0%) agreed that AMR could become a problem in their animals. Only 19.3% of farmers disagreed that ABU in animals should be reduced. Factors influencing practices are summarised in Fig. 9. Disease symptoms in animals prompted 92.4% of farmers to use antibiotics. Increases in mortality and morbidity, and reduction in animals’ appetites were also “Most Likely” or “Likely” to trigger antibiotic use for most farmers. More than three-quarters (77.6%) indicated they were “Most Likely” (19.9%) or “Likely” (57.7%) to use antibiotics as a means of improving the growth rate or productivity of their animals, and 77.2% were “Most Likely” (18.2%) or “Likely” (59.0%) to use antibiotics prophylactically. The factors least likely to influence farmers’ antibiotic use were cost and availability.

Bivariate analysis indicated that the herd size was the only demographic variable not significantly associated with the KAP of farmers (Supplementary Table 2). In the multivariate model, farmers aged 60 years or older were 24 times more likely to have higher knowledge than farmers younger than 20 years (AOR: 23.7, 95% CI: 2.7–560.0, *p*: 0.013) (Table 5). Farmers from Niger State were 3 times more likely to have higher knowledge scores than farmers from Kwara State (AOR: 2.9, 95% CI: 1.9–4.4, *p* < 0.001). Niger State farmers were also four times as likely to have higher practice scores (AOR: 4.0, 95% CI: 2.7–6.1, *p* < 0.001) but 83% less likely to have better attitudes than Kwara State farmers. Farmers with secondary and tertiary levels of education were likely to have higher knowledge scores when compared to farmers with no formal education. Spearman’s correlation indicated that all categories (knowledge, attitudes, and practices) were positively correlated with each other (*p* < 0.001).

**Table 5:** Multivariate analysis for KAP

Variables	Category	Knowledge		Attitudes		Practices	
		AOR* (95% CI)	p-value	AOR (95% CI)	p-value	AOR (95% CI)	p-value
Age (years)	≤ 20	r**		r		r	
	21–30	7.57 (1.01–166.00)	0.092	0.71 (0.14–4.20)	0.688	8.34 (1.29–165.00)	0.059
	31–40	5.11 (0.69–111.00)	0.172	0.75 (0.15–4.36)	0.728	8.90 (1.39–176.00)	0.051
	41–50	9.12 (1.22–200.00)	0.066	0.74 (0.15–4.36)	0.719	8.28 (1.27–164.00)	0.060
	51–60	8.59 (1.12–191.00)	0.076	0.94 (0.18–5.71)	0.945	8.49 (1.27–170.00)	0.059
	> 60	**23.70 (2.68–560.00)**	**0.013**	1.49 (0.24–10.80)	0.676	4.32 (0.55–92.6)	0.222
Gender	Male	r		r		r	
	Female	1.08 (0.72–1.61)	0.724	0.74 (0.48–1.15)	0.186	0.94 (0.63–1.39)	0.761
State	Kwara	r		r		r	
	Niger	**2.90 (1.93–4.39)**	**< 0.001**	**0.17 (0.11–0.25)**	**< 0.001**	**4.02 (2.69–6.08)**	**< 0.001**
Level of Education	No formal education	r		r		r	
	Primary School	1.28 (0.78–2.10)	0.318	0.80 (0.47–1.37)	0.421	**0.54 (0.32–0.88)**	**0.014**
	Secondary School	**2.70 (1.70–4.32)**	**< 0.001**	0.65 (0.39–1.09)	0.102	0.86 (0.54–1.37)	0.524
	Tertiary Education	**2.40 (1.38–4.20)**	**0.002**	0.59 (0.32–1.11)	0.106	0.69 (0.39–1.19)	0.179
Role on Farm	Owner	r		r		r	
	Owner and manager	0.94 (0.65–1.36)	0.756	**1.63 (1.12–2.38)**	**0.012**	**1.46 (1.03–2.07)**	**0.032**
	Manager	**2.01 (1.07–3.82)**	**0.031**	1.16 (0.58–2.29)	0.677	2.04 (0.85–5.21)	0.192
	Worker	**4.33 (1.69–12.80)**	**0.003**	0.51 (0.13–1.53)	0.275	0.65 (0.33–1.23)	0.121
Number of years in ruminant farming	≤ 10	r		r		r	
	11–20	1.38 (0.92–2.09)	0.121	1.49 (0.97–2.31)	0.070	0.68 (0.46–1.00)	0.052
	21–30	1.34 (0.76–2.40)	0.315	1.78 (0.99–3.19)	0.054	0.82 (0.47–1.42)	0.482
	31–40	1.27 (0.76–2.40)	0.501	0.76 (0.38–1.52)	0.438	1.23 (0.63–2.43)	0.540
	> 40	0.90 (0.32–2.44)	0.846	0.85 (0.32–2.31)	0.743	1.97 (0.79–4.93)	0.145

Bolded AORs and p-values indicate statistical significance

*AOR Adjusted odds ratio **r reference category

### Factors influencing farmers’ treatment decisions and use of antibiotic and ATAs

**Fig. 10:**
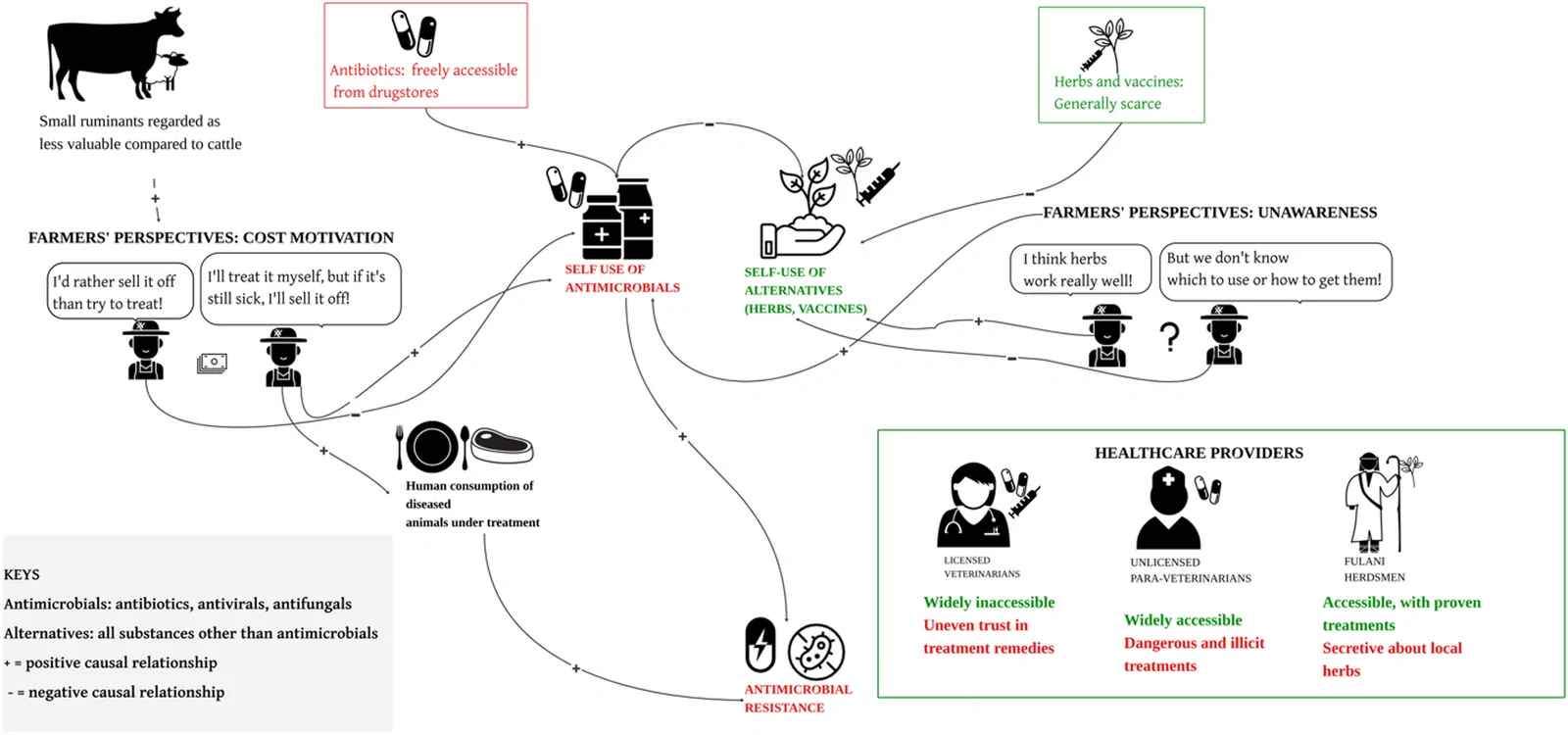
Factors influencing farmers' treatment decisions as uncovered in the focus discussions (rich picture diagram)

Themes derived from the FGDs covered economics, effectiveness, and availability. Figure 10 summarises the interactions of these factors and how they influence farmers’ health management decisions. The themes are discussed below:

#### Economic pragmatism outweighs animal health considerations in farmers’ health management decisions

Many small ruminant farmers also kept a cattle herd. As cattle had higher monetary value, farmers tended to prioritize and treat disease outbreaks within this herd. Small ruminants, however, were more likely to be sold off after farmers observed diseases among them. A farmer said the following when comparing cattle to small ruminants:

*“Anyone [who is] rearing cattle has to be very careful [with their health management practices]. You can take risks on sheep and goats but not cattle.” – R05*,* small ruminant farmer.*

One farmer remarked on the difference in cost value after diseases between the two species here:

*“Earlier this year we had two cows. If they were healthy*, *they are worth about* ₦*800*,*000 each*, *but they were affected by tick infestation. We later sold them at* ₦*450*,*000 […] For ram*, *if it was worth at least* ₦*100*,*000*, *we can mostly sell it at* ₦*40*,*000 [after dermatophilosis].” - R12*,* small ruminant farmer.*

Some diseases, such as mastitis, were considered not to affect the cost of sale in a significant manner:

*“[Mastitis] doesn’t actually affect the cost of sale like you think. [The cost of sale] is only affected when the [disease is evident] on the body. When the [symptoms are internal]*,* and you buy [the animal] and flame it [before cutting it up for retail sale]*,* [the disease] will not show. During [slaughter]*,* if we see [evidence] of mastitis*,* we can cut [the teat] out. I can’t really specify [how much loss might be incurred then] […] It could be a 25% loss.” - R02*,* animal seller/butcher.*

#### Trust and perceived effectiveness drive farmers’ use of treatment options and health networks

While some farmers used veterinarians, most farmers primarily relied on themselves to manage diseases due to the unavailability of licensed veterinarians and a lack of trust in their prescribed treatments, as stated here:

*“I don’t consult doctors. I have consulted before but there was no solution to our problem*,* so I go on self-medication.” – R04*,* small ruminant farmer.*

Para-veterinarians, though widely used, were similarly distrusted due to their penchant for providing illicit, expired, or potentially dangerous mixtures. One farmer said:

*“I have seen [a paraveterinarian] mixing tetracycline with petrol and injecting it in the lung [of a cow]. Within 48 hours*,* 60 of the cows were [dead].” - R13*,* small ruminant farmer.*

However, Fulani herdsmen (ruminant farmers with a traditionalist pastoral lifestyle who were relied on by farmers as herbalists) were trusted by farmers for their traditional herbal remedies:

“*There was a case of lameness [on my farm] that I doubted the Fulani man but the next morning [after the Fulani herbalist used some herbs to treat the animal]*,* the animal [could stand]. So I believe it works. We want [good] results and we are getting it. That is all that matters.” - R12*,* small ruminant farmer.*

While using antibiotics and ATAs personally, farmers were split regarding their perceptions of the use of antibiotics and ATAs. Some farmers believed that herbs were more effective than antibiotics, while some farmers thought the opposite. Two farmers said:

*“I don’t use antibiotics and we get [good] results anytime I use the herbs.” - R03*,* small ruminant farmer.*

*“The herbal remedies are not as effective as before [for mastitis].” - R18*,* small ruminant farmer.*

#### Availability, access and information affect farmers’ use of antibiotics and ATAs

Antibiotics were widely used by farmers because they were readily available from unlicensed drug stores. Farmers who distrusted the validity of drugstore supplies could also procure them from licensed veterinary stores. One farmer said:

“*We use antibiotics [on our farm]. We get it from reputable veterinary outlets not [drug store vendors] and street hawker*s.” - *R16*,* small ruminant farmer.*

Conversely, some farmers could not easily use herbs because they were not easy to procure:

*“Our major problem with the herbs is the scarcity of the [herbs]. I prefer it to conventional [antibiotics]*,* but sometimes I don’t have access to them easily.” - R09*,* small ruminant farmer.*

Many farmers also did not know which plants were effective herbs for specific conditions. This caused farmers to rely on Fulani herdsmen (traditional pastoralists skilled in herbal preparations) for consultations. However, Fulani herdsmen were usually secretive about the herbs. One farmer said:

“*I do [use herbs]. I get it from the herdsman (Fulani)*,* but he won’t disclose the concoction content” - R12*,* small ruminant farmer.*

The lack of knowledge about effective herbs and their dosages affected farmers’ use of them:

*“The problem is the dosage of these herbs [which I don’t know].” - R05*,* small ruminant farmer.*

Veterinarians were also considered unavailable and inaccessible, compared to para-veterinarians and drugstore vendors, who were widespread:

*“Before*,* [farmers in our community] used to call vet doctors that moved around (doing ambulatory services). But now*,* many farmers don’t know [any] vet doctors. [Vet doctors] have not come [to our community] since 15 years ago. Now*,* just anybody sells the drugs. Some are not even educated*,* and they are selling the drugs*,* because there is no doctor.’ - R15*,* small ruminant farmer.*

## Discussion

Our survey investigated the production characteristics of small ruminant farmers in North-Central Nigeria, their health management practices, and possible factors influencing the use of antimicrobials and ATAs. The majority of small ruminant farmers were male and owned less than 50 animals in their stock. Similar reports on gender distribution and flock size have been observed in other Nigerian small ruminant studies [3, 31, 32]. However, our finding contrasts with other studies that identify women as primary actors in small ruminant rearing, particularly on a household level [33]. It should be noted that our study primarily recruited people who engaged in small ruminant farming as a commercial venture rather than for subsistence only. While women are also involved in ruminant farming in this regard, societal constraints often limit their ability to participate to the same extent as men [32, 33].

Our findings indicated that most farmers kept a mixed flock rather than sheep or goats alone. Sheep and goats have varying disease susceptibility. Goats are more susceptible to PPR than sheep, and mastitis prevalence is often found to be higher in the former [9, 34]. CCPP is also primarily a disease of goats [35]. Farmers mostly used an open housing system without netting. Within this housing system, animals may be attacked more frequently by insects and, therefore, are more susceptible to insect-borne infections such as dermatophilosis and trypanosomiasis [36, 37].

Diseases affecting small ruminants as observed by farmers in our study included mastitis, PPR and dermatophilosis. While other studies have similarly documented the high prevalence of these diseases, additional evidence suggests that there are several more endemic conditions, including CCPP and helminthiasis, affecting small ruminants in Nigeria [9, 11, 38, 39]. Likely, these diseases occur frequently within farms surveyed; however, farmers may not have reported them due to misdiagnosis. As farmers rely mostly on physical observation to diagnose, CCPP may be mistaken for PPR due to similarities in their clinical presentations [9, 39]. The misdiagnosis of bacterial diseases as viral diseases, or vice versa, may lead to antimicrobial misuse, potentially contributing to the high levels of antimicrobial misuse observed among Nigerian ruminant farmers [12]. In addition, some diseases may be overlooked entirely. For instance, subclinical mastitis (SCM) is the most common form of mastitis affecting small ruminants in Nigeria, but farmers in this survey did not account for it [11]. This is likely because SCM does not present with physical lesions but rather through poor productivity and performance [11, 38]. Flocks likely suffer from more health issues than farmers are currently aware of. This suggests consistent flock underperformance and unquantified losses to diseases.

Our results indicated that preventive measures were rarely adopted on farms for mastitis, and low vaccination rates were generally observed for CCPP and PPR. In 2024, the Federal Government of Nigeria launched a PPR vaccination campaign as part of the PPR Global Eradication Programme (PPR - GEP) by the Food and Agriculture Organisation (FAO) [40, 41]. This might explain the higher vaccination rates noticed for PPR. Still, only 27% of surveyed farms had received the PPR vaccine. According to our findings, farmers mainly rely on government veterinarians to access vaccines; thus, low vaccination rates likely indicate low reach by governmental vaccination efforts. The high proportion of farmers who did not implement preventive practices against mastitis is not surprising, as most farmers were unaware of the causes of mastitis. Therefore, it would be difficult for farmers to know what measures to implement. Our finding aligns with a previous study that noted a lack of adoption of mastitis preventive measures among Nigerian dairy farmers [21]. Much progress can be achieved in preventing diseases if farmers are equipped with the right tools (vaccines, information, and veterinary services) for protecting their flocks. One case study of this is the 2012 “DairyCo Mastitis Control Plan (DMCP),” a United Kingdom (UK) structured plan to reduce the prevalence of bovine mastitis. Here, veterinarians were trained to go to farms and design targeted intervention plans. This led to a more than a 20% drop in mastitis cases [42, 43]. The potential of using a similar model for small ruminant mastitis in Nigeria could be assessed, while methods to improve vaccine reach for CCPP and PPR are investigated.

Antibiotic use was self-guided and unconstrained among farmers. A previous study on North-Central Nigerian pastoralists observed that most farmers practiced self-administration, and only 27% of farmers followed the label instructions [12]. Similarly, findings from a USA study noted that 80% of small ruminant farmers self-administered antibiotics on their farms [44]. Antibiotic classes commonly used included tetracyclines, penicillins, and macrolides. Similarly, these antibiotics have been reported to be commonly misused in small ruminant farms in Nigeria and the USA [12, 44]. All of these antibiotic classes have been classified as Veterinary Critically Important Antimicrobial Agents (VCIAs) by the World Organisation for Animal Health (WOAH), due to the scarcity of potent therapeutic substitutes when resistance to these antibiotics arises [45]. In addition, macrolides were classified as Critically Important Antimicrobials (CIA) in humans by the World Health Organisation (WHO) [46]. The misuse of these antibiotics in small ruminants bears significant consequences for animal and human health, as ARGs can be transmitted between animals and from animals to humans through risk pathways of environmental contamination and consumption of contaminated food products [12, 46, 47].

Farmers’ KAP regarding antibiotics and AMU is of concern and is reflected in the high percentages of farmers who are ‘very likely’ or ‘likely’ to use antibiotics for growth promotion and to boost productivity. Most farmers had not heard of the term ‘AMR’ and did not think AMU in animals should be reduced, even though 41.8% believed that antibiotics were no longer effective. This indicates that farmers are starting to observe a growing problem with AMR, but there is generally low awareness about how AMR develops or methods to curtail it. Similarly, an Ethiopian study found that 56% of animal producers were not aware that their AMU contributed to AMR [48]. Dewi et al. [49] also found that only 35% of farmers in an Indonesian province were aware that over- and underdosing during AMU contributed to AMR. Currently, Nigeria’s livestock AMU is expected to increase by 163% before 2031 [50]. There is a need for urgent practice reform within Nigerian small ruminant farming to improve AMU practices, and studies elsewhere show that education programmes can have a dramatic influence. A study in France found that cephalosporin use in swine production fell by 90% in the five years following farmers’ enrolment in an antimicrobial stewardship (AMS) programme [51]. As observed in our study, and higher knowledge of AMU and AMR translated to better attitudes and practices. This indicates that raising awareness among Nigerian farmers about antimicrobial misuse may improve their current practices.

Herbal plants and household materials were used for health management, such as the use of *Khaya senegalensis* and beans in treating mastitis. Chah et al. [22] observed that small ruminant farmers in Eastern Nigeria used *Spondias monbin* for the treatment of mastitis while also employing various herbs in managing diarrhoeal symptoms from PPR. Conversely, none of the farmers interviewed mentioned being aware of this herb. This indicates that plants used in small ruminant farming may differ by region in Nigeria, highlighting the extensive diversity of herbs in the country. Similar to disease diagnosis, farmers appeared to use ATAs primarily for alleviating physical symptoms, such as the hardness of the teat observed in clinical mastitis. Thus, farmers may consider an animal healed while the animal is still infected and manifesting less obvious symptoms. Still, herbs identified by farmers have been recorded to possess antibacterial activities. *Khaya senegalensis* has been proven to inhibit the growth of bacterial isolates during lab-based studies, including *Staphylococcus aureus and Escherichia coli*, implicated in small ruminant mastitis [52, 53]. Likewise, *Vernonia amygdalina* has also exhibited antibacterial activity even against multidrug-resistant bacterial isolates [54]. However, very few studies have been conducted to determine the mechanisms of action of these drugs against small ruminant diseases, nor the effective therapeutic dosages required. Thus, more research and development (R&D) efforts are indicated in these areas.

Cost affected farmers’ decisions regarding treatment, with some farmers selling off ill animals or animals currently undergoing treatment. These practices have been banned in countries like the United Kingdom to protect human health from possible zoonotic transmission [55]. Similarly, Sect.  247 of the Nigerian Criminal Code prohibits the sale of diseased animals for human consumption [56]. Our finding indicates that these laws are weakly enforced. Due to economic considerations, farmers were less reluctant to expend efforts in treating small ruminants when compared to cattle. This is not surprising, as FGD participants estimated the value of a diseased cattle to be four times higher than that of healthy small ruminants. As ATAs, particularly herbal plants, are the cheapest form of treatment available, cost-saving interests likely motivate farmers to use them rather than antibiotics for managing small ruminant diseases. Likewise, Chah et al. [22] observed that farmers stated they used herbal plants as therapeutic agents due to their being low or no cost.

We observed that most farmers did not use ATAs, citing reasons of low access and availability. Most farmers were unaware of specific plants effective for diseases, where to procure them from, and what dosages to use. These reasons likely contributed to why far fewer farmers (14%) used herbs over the last three months when compared to farmers who used antibiotics (48%). Similarly, a previous study reported that 43% of livestock farmers in Osun State, Nigeria, did not use herbal remedies due to poor knowledge of herbs and their application [23]. In our study, farmers relied on the expertise of Fulani herdsmen when unaware of how to use the herbs. The Fulanis are Northern Nigerians with a traditional pastoralist lifestyle and a culture of preserving ethnoveterinary knowledge through verbal communication across generations [57, 58]. However, Fulani herdsmen require payment for their consulting services and were reported by farmers to be generally secretive about the herbs used. Without relying on Fulani herdsmen, farmers appear to be unaware of the full assortment of herbs available in their community, proper use practices, and their efficacy against endemic conditions. Still, less than 5% of farmers consulted herbalists (including Fulani herdsmen) for treatment services within the last three months. It is likely that farmers’ personal unawareness about the herbs reduced their interest in using this enterprise, and consequently their dependence on herbal remedies.

Trust in veterinarians was uneven and shaped by farmers’ perceptions of their treatments being effective. In a study conducted in India, veterinarians stated that farmers only consulted them after trying multiple treatment options and when AMR had set in [59]. Likewise, farmers here stated that the most significant factor influencing their antibiotic prescription was the observation of disease symptoms in their flock, rather than advice from veterinarians. Therefore, farmers likely consulted veterinarians after self-treatment had failed, leading to veterinarians being unlikely to succeed at treatment using antimicrobials due to the development of resistance. Possible reasons identified for farmers’ delay in consulting veterinarians include an intent to save costs and the general unavailability of veterinarians. Currently, veterinarians are relatively scarce in Nigeria, with an estimated ratio of 1 veterinarian per 37,500 animals [60, 61]. Due to the lack of veterinarians, more farmers tended to consult with unqualified para-veterinarians. Other studies have reported that para-veterinarians are a regular feature in low- to middle-income countries where qualified veterinary services are scarce, as they are widespread and charge less for consultation [59]. While farmers stated they distrusted them, para-veterinarians were still the most frequented animal healthcare service provider, as they were the most available. It is crucial to examine and address the lack of qualified animal healthcare professionals for small ruminants in Nigeria, as this is a significant motivator for self-prescribed AMU, and consequently rising AMR risk.

### Study limitations

Disease prevalence estimates were not determined through epidemiological surveys and laboratory analysis but were based on farmers’ reports. Therefore, figures may be affected by memory recall and reliance on physical observation for diagnosis. This might have caused misdiagnosis of several diseases. However, this approach was used to help understand small ruminant farmers’ perceptions of prevalent diseases and their health management practices.

## Conclusions and recommendations

There was a general dependence on self-prescribed antibiotics over vaccines and herbal ATAs among small ruminant farmers in North-Central Nigeria. Consequently, AMU was indiscriminate and bore grave implications for AMR. There is a need to improve access for farmers to vaccines, government veterinarians, and laboratory diagnostic services. It is also essential to curtail the unlicensed sale of veterinary drugs through the enforcement of antimicrobial stewardship (AMS) measures. Future initiatives can focus on: (1) improving farmers’ awareness of AMR and its risk practices; (2) conducting awareness programmes to sensitize farmers about effective disease preventive measures and the importance of maintaining proper hygiene and sanitary conditions; (3) evaluation of the current vaccine distribution channels in Nigeria and how to improve delivery and reach; (4) research on available herbal remedies in Nigeria and assessing their effectiveness against prevalent small ruminant diseases; (5) exploring barriers that hinder better compliance with antimicrobial regulations.

## Supplementary Information

Supplementary Material 1. Supplementary Figure 2

Supplementary Material 2. Supplementary File 1

Supplementary Material 3. Supplementary Table 1

Supplementary Material 4. Supplementary Table 2

Supplementary Material 5. Supplementary File 2

Supplementary Material 6. Supplementary Figure 1

## Data Availability

The data that support the findings of this study are obtainable from the corresponding author upon reasonable request.
