# Supplementary material for: Small ruminant production system characteristics and their influence on use, alternatives of, and resistance to antimicrobials in Nigeria
Source: BMC Vet Res. 2026 Jun 20;22:602. doi: 10.1186/s12917-026-05577-7 (PMC13663043; doi:10.1186/s12917-026-05577-7)
Supplement: Supplementary file 1 [file 12917_2026_5577_MOESM2_ESM.docx]

**Additional File 1: Small Ruminants Farm Health Management Questionnaire**

**Section 1: QS details**

| Survey code: | __/_ _/_ _ _/_ _ _ | (State/LGA/Village/Farm Sn) |
| --- | --- | --- |
| Interviewer’s name: | First name | Family name |
| Interview date: | _ _/_ _ /_ _ _ _ (DD/MM/YEAR) | |
| Consent obtained: | 1=Yes 2=No *(if no, request a replacement herd from the field work team leader)* | |

**Section 2: Interviewee details**

| Age (years): | 1= less 20, 2=21-30, 3=31-40, 4=41-50,  5=51-60 6=Greater than 60 |
| --- | --- |
| Interviewee’s gender: | 1=Male 2=Female 3=Other |
| Level of education: | 1= No formal education,  2= Primary school,  3= Secondary School  4= College/Polytechnic/University  5= Postgraduate  6= Other: Specify_________________________________________) |
| What is your role in the herd? | 1 = Owner 2 =Manager 3 = Owner and manager  4 = Worker 5=Other: (Specify___________________________) |
| No. of years in small ruminants rearing? /Years | |

**Section 3: Herd characteristics**

1. How many animals do you have in this herd?

|  | Total No. per breed | Age | | | | | |
| --- | --- | --- | --- | --- | --- | --- | --- |
|  |  | Less than 6 months | | 6 months to 2 years | | More than 2 years | |
|  |  | M | F | M | F | M | F |
| Sheep |  |  |  |  |  |  |  |
| Goat |  |  |  |  |  |  |  |

2. For animals more than 1 year old, please complete the following table, how many animals are there?

| Animal status | Number |
| --- | --- |
| Milking/lactating |  |
| Dry |  |
| Pregnant |  |

3. For lactating animals, what is the average milk production per dam per day?

| Season | Amount |
| --- | --- |
| Wet |  |
| Dry |  |
| Average |  |

4. For lactating animals, what is the average milk production per dam per day? _____/L
5. What is the average daily milk produced in this herd over the last year (*please ask for the milk record and have a look*)? __________/L a day
6. What do you do with the milk produced at your herd?

| Used for | Average quantity per day/L |
| --- | --- |
| Home consumption as fresh milk |  |
| Homemade dairy products (e.g. cheese) |  |
| Raw milk sold to milk collectors |  |
| Raw milk sold directly to consumers |  |
| Pasteurize before selling |  |
| Others |  |

7. Other than animals born at your herd, do you buy **new/replacement** animals for rearing at your herd?

1=Yes	2=No	(*If No Please go to QS 9)*

8. **Over the last 12 months** how many animals have you purchased? *(Please complete the following table for* ***newly purchased*** *animals)*

| Age/months | Gender | Reason for purchase |
| --- | --- | --- |
|  |  |  |
|  |  |  |
|  |  |  |
|  |  |  |
|  |  |  |

9. Do you sell any animals from your herd?

1=Yes	2=No	*(If No Please go to QS 11)*

10. **Over the last 12 months** how many animals have you sold/culled? *(Please complete the following table for* ***sold animals****)*

| Age/months | Gender | Reason for sale/cull |
| --- | --- | --- |
|  |  |  |
|  |  |  |
|  |  |  |
|  |  |  |

**Section 4: Herd Management**

11. What type of housing is used in this herd?

1=Closed housing system	2=Open housing system with netting

3=Open housing system without netting 4= Others (	)

12. Feeding practices?

1=Extensive (Grazing only)	2= Semi-intensive (Grazing and stall feeding)

3=Intensive use TMR (Total Mixed Ration)

13. In case of grazing and stall-feeding, how many hours of grazing per day?

1=2-4 hrs	2=4-6 hrs	3=6-8 hrs	4=Others (	)

14. What are the main types of feed do you use for your herd? (select all that apply)

1=Natural pastures	2= hay	3= Corn silage

4= Concentrate (corn, barley, dried distillers grains with soluble (DDGS), oil seed meals)	5= Commercial premixed feed	6=Crop residues (e.g., straws)

15. In semi-intensive production, how many kg/day of feed is given for each cow?

1= 2 kg feed	2= 4 kg feed	3=6 kg	4=others (	)

16. Do you have a grazing land/field for your herd?

1=Yes, 2= No, 99= Don’t know

17. Do you use a community/public/random grazing fields for your animals?

1=Yes, 2= No, 99= Don’t know

**Section 5: Animal Health Management**

18. Which **records** do you keep for this herd?

| Record for | 1=Yes, 2= No, 99= Don’t know |
| --- | --- |
| Feed |  |
| Milk |  |
| Veterinary health products (treatment/drugs) |  |
| Reproduction |  |
| Vaccination programmes |  |

19. Do you **vaccinate** your herd against diseases?

1=Never	2=Sometimes	3=Always	*(If No Please go to QS No 21)*

20. If you vaccinate your herd, please complete the following table (*please use one raw for each type of vaccine*).

| Vaccine against (name of the disease) | Number of times/year | Vaccine provider/Source of Vaccine | Cost per animal per dose |
| --- | --- | --- | --- |
|  |  |  |  |
|  |  |  |  |
|  |  |  |  |
|  |  |  |  |

21. How do you know that you have **a health problem in your herd**? *(Allow multiple)*

1=Experience	2=Other herders experience	3=Changes in animal behaviour

4=Increasing mortality rate	5=Private doctor veterinarian

6=Government veterinary officer	7=Private para-vet

8=Others (Specify_________________________________________)

22. Over the **last 3 months**, what are the health problems did you have in this herd?

| Health problem | 1=Yes 2=No | What did you do? |
| --- | --- | --- |
| Gastrointestinal |  |  |
| Respiratory |  |  |
| Lameness |  |  |
| Reproductive |  |  |
| Mastitis |  |  |
| Others |  |  |

23. What do you do when you **suspect a health problem** in your herd (please list what you do in a ranking order, what do you do first? If it doesn’t work what do you do)?

…………………………………………………………………………………………………

…………………………………………………………………………………………………

24. What do you do to protect your dairy animals from **mastitis**?

…………………………………………………………………………………………………

…………………………………………………………………………………………………

25. Do you check all milking animals (in one milking) for mastitis?

1=Yes, 2= No, 99= Don’t know

26. If yes, how frequently do you check all milking animals (in one milking) for mastitis?

1=Daily	2=Weekly	3=Monthly	4=Annually

5=When there is a high somatic cell count

6=Others (_____________________________________)

27. In your opinion what are the causes of **mastitis** in your herd?

…………………………………………………………………………………………………

…………………………………………………………………………………………………

…………………………………………………………………………………………………

…………………………………………………………………………………………………

28. Over the last 12 months, did you have any case of **mastitis** on your herd?

1=Yes, 2= No, 99= Don’t know

29. If yes, how many cases of **mastitis** did you have over the last 12 months? _________
30. Over the last 12 months, what was the most common form of **mastitis** on your herd?

1=Clinical mastitis	2=Subclinical mastitis	99= Don’t know

31. What do you do when you suspect a case of **mastitis** in your herd (The first action you do)?

…………………………………………………………………………………………………

…………………………………………………………………………………………………

32. What if the treatment did not work, what did you do?

…………………………………………………………………………………………………

…………………………………………………………………………………………………

33. What do you do when you suspect a case of mastitis in your herd, would you start treatment on your own?

1=Yes, 2= No, 99= Don’t know

34. If your answer is yes, what kind of treatment do you apply?

……………………………………………………………………………………………………………………………………………………………………………………………………

35. Would you start antibiotic treatment without asking the veterinarian?

1=Yes, 2= No, 99= Don’t know

36. If yes, which antibiotic do you use? ……………………………………………………………………………………………………………………………………………………………………………………………………
37. Do you cull animals due to persistent mastitis?

1=Yes, 2= No, 99= Don’t know

38. What methods are you currently using to detect **mastitis** in your herd? *(Select all that apply)*

1=Observation	2=California mastitis test	3=Milk somatic cell count

4=Conductivity test	5=Others (please specify______________)

39. Do you or your veterinarian take samples for laboratory diagnosis of **mastitis**?

1=Never	2=Sometimes	3=Always

40. Which samples were collected?

…………………………………………………………………………………………………

…………………………………………………………………………………………………

41. Where did you analyse these samples?

1=On-herd	2=Private lab	3=Government lab	4=Others (_________________)

42. How much did it cost you to analyse one milk sample in a laboratory? _____ NGN/Sample
43. How long does it take from sampling to results? _______________/hs
44. Do you or your veterinarian use on-herd diagnostic tools for mastitis on your herd?

1=Yes, 2= No, 99= Don’t know

45. If Yes what are these tools?

…………………………………………………………………………………………………

…………………………………………………………………………………………………

46. How much did it cost you to analyse one milk sample on-herd? _____ NGN /Sample
47. How long does it take from sampling to results? _______________/hs
48. For one case of **mastitis** in one quarter, what is the average cost of medications? ____ NGN
49. For one case of **mastitis** in one quarter, what is the average cost of veterinarians? ___ NGN
50. For one case of **mastitis** in one quarter, what is the average cost due to milk loss? ___ NGN
51. Have you heard about **mastitis** in cattle caused by a resistant pathogen?

1=Yes, 2= No, 99= Don’t know

52. If yes, how did you know?
53. Have you used any **animal health services** in the last 3 months?

1= Yes	2= No	*(If No Please go to QS No 55)*

54. If yes, please complete the following table using the following codes: (***Type of service****: 1= vaccination; 2= other preventive methods, specify which one, 3= treatment of sick animals; 4= other, specify, S****ervice Provider:*** *1= official/Government veterinarian, 2= Private veterinarian, 3=Paravet, 4=Traditional/herbal medicine 5= Pharmacist 6= Community veterinary health provider 7=Other specify_________________*)

| Type of services | Service provider | Do you pay for the service (1=Yes, 2=No) |
| --- | --- | --- |
|  |  |  |
|  |  |  |
|  |  |  |
|  |  |  |
|  |  |  |

55. From where do you get/buy **veterinary health products and feed additives as vitamins/minerals**?

| **Source of products** (1= Veterinary drug store, 2=Veterinarian doctor, 3=Para-vet, 4=Government veterinary clinic, 5=Human pharmacy, 6=Animal feed store, 7=Other (Specify__________) | **Importance** (1= Not important; 2=Slightly important; 3=Fairly important; 4=Important; 5 =Very important) |
| --- | --- |
|  |  |
|  |  |
|  |  |
|  |  |
|  |  |

56. What is the source of information for the **veterinary health products** that you used for your herd?

| **Source of information** (1= Other herders, 2=Feed technician, 3=Product supplier, 4=Government veterinary doctor, 5=Private veterinary doctor, 6= Private para-vet, 7=Prior experience, 8=Dairy association, 9=Other (Specify__________) | **Importance** (1= Not important; 2=Slightly important; 3=Fairly important; 4=Important; 5 =Very important) |
| --- | --- |
|  |  |
|  |  |
|  |  |
|  |  |
|  |  |

**Section 6: Knowledge and Attitudes towards Antibiotics and Antibiotic Resistance (ABR)/Antimicrobial Resistance (AMR)**

57. Have you heard about the term **antibiotics**? *(Please ask to see the packages or any evidence for the product also show him/her the list of products you have and ask him/her if he/she has used it, please add more lines as needed, they might be using antibiotics but they don’t know)*

1=Yes, 2= No, 99= Don’t know

58. In your opinion, what are **antibiotics** used for? *(Allow multiple answers)*

1=Increasing animal growth and production, 2=Protecting against diseases in healthy animals, 3=Treating diseased animals, 4=Others (Specify ______________________)

59. Over the **last 3 months**, have you used **antibiotics** for your herd?

1=Yes, 2= No, 99= Don’t know	*(If No Please go to QS No 62)*

60. If yes, please complete the following table. *(Please ask to see the packages or any evidence for the product also show him/her the list of products you have and ask him/her if he/she has used it, please add more lines as needed)*

| Antibiotic name | Health Problem Description | Cost per case |
| --- | --- | --- |
|  |  |  |
|  |  |  |
|  |  |  |
|  |  |  |
|  |  |  |

61. For the above list of products, please complete the following table.

| Antibiotic name | Route of administration (1=Oral, 2=Injection, 3= Others) | Number of days used |
| --- | --- | --- |
|  |  |  |
|  |  |  |
|  |  |  |
|  |  |  |
|  |  |  |

62. What do you do with the antibiotic leftover?

……………………………………………………………………………………………….

63. What do you do with the milk from animals under treatment with antibiotics?

…………………………………………………………………………………………………

64. If you didn’t use **antibiotics** what would happen to your animals?

……………………………………………………………………………………………………………………………………………………………………………………………………

65. Do you think there is an economic advantage in using **antibiotics**?

1=Yes, 2= No, 99= Don’t know	*(If No Please go to QS No 67)*

66. If yes, why?

……………………………………………………………………………………………………………………………………………………………………………………………………

67. Do you think **antibiotics** are effective?

1=Yes, 2= No, 99= Don’t know

68. Which of the following factors are likely to influence your decision to use **antibiotics** for your herd animals? (*Please read out each factor and ask him/her to respond by 1=Not likely, 2=Likely, 3=Most likely*)

| Factor | Responses |
| --- | --- |
| Presence of disease sign/symptoms on the animal/s |  |
| To protect animals from getting infected “healthy animals and no symptoms for disease” |  |
| The morbidity rate has increased |  |
| The mortality rate has increased |  |
| The animals are not eating |  |
| To improve the productivity and growth of animals |  |
| A drug seller advises that antibiotics are needed |  |
| A veterinarian/animal health professional advises that antibiotics are needed |  |
| Para veterinarian advises that antibiotics are needed |  |
| Another herder advises that antibiotics are needed |  |
| Availability of antibiotics |  |
| Costs of antibiotics |  |
| Other factors (___________________________________) |  |

69. Who is responsible for prescribing **antibiotics** for your herd animals?

1=Doctor Veterinarian, 2=Para-vet, 3=Myself, 4=Another family member

5=Others (Specify__________________________________________)

70. If you have used an **antibiotic** and it was not effective, what would you do?

1=Increase the dose of the same **antibiotic**	2=Use another **antibiotic**

3=Use a combination of more than one **antibiotic**

4=Consult the Doctor-vet or the Paravet again

5=Others (____________________________________)

71. In your opinion, would it be possible to raise animals without using **antibiotics**?

1=Yes, 2= No, 99= Don’t know

72. Do you know any alternatives for **Antibiotics**?

1=Yes, 2= No, 99= Don’t know	*(If No Please go to QS No 74)*

73. What are the alternative products for **antibiotics** do you know?

………………………………………………………………………………………………………………………………………………………………………………………………………………………………………………………………………………………………………

74. Do you use any of the **antibiotic** alternative products?

1=Yes, 2= No, 99= Don’t know	*(If No Please go to QS No 76)*

75. If yes, please list the **antibiotic** alternative products you use. *(Please ask to see the packages or any evidence for the product also show him/her the list of products you have and ask him/her if he/she has used it)*

………………………………………………………………………………………………………………………………………………………………………………………………………………………………………………………………………………………………………

76. If no, why don’t you use alternative products?

1=Expensive compared with antibiotics	2=Not easily available	3=Not effective

4=Difficult to administer	5=Others (____________________________)

77. Have you ever heard about a term called **“Antibiotic Resistance (ABR)/Antimicrobial Resistance (AMR)”**?

1=Yes, 2= No, 99= Don’t know	*(If No Please go to QS No 79)*

78. If yes, from whom have you heard about **ABR/AMR**?

1=Doctor Veterinarians	2=Para-vet	3=Government department of animal health	4=Government department of human health	5=Neighbours/friends

6=Dairy associations/federations	7=Media

79. Could you please respond to the following statement, using (1= disagree, 2= Neutral, 3= Agree and 99=Don’t know)? *(Please read out all statements below and record the responses)*

| Statement | Response |
| --- | --- |
| ABR/AMR will never be a problem on my herd animals |  |
| The more **Antibiotics** I use on my animals the healthier they are |  |
| My personal health is positively affected by using **Antibiotics** in animals |  |
| **Antibiotic** use in animals can negatively affect the health of consumers |  |
| **Antibiotic** use in animals should be reduced |  |
| **Antibiotic** use in humans should be reduced |  |
| Overuse of **antibiotics** can lead to antibiotic resistance |  |
| Many **Antibiotics** are not effective for treating diseased animals |  |
| Improper use of **Antibiotics** results in high costs for herders |  |
| Using **Antibiotics** in animals may affect their effectiveness in humans |  |

80. Over the last year, have you attended any of the following training?

| Training *(please note this list is indicative, ask for any other training)* | 1=Yes 2=No | Training provider (1=Feed company, 2=Dairy processing company, 3=Pharmaceutical company/supplier, 4=Government organisation,  5= Others ______) | Satisfaction (5=Very high, 4=High, 3=Moderate, 2=Low,  1=Very low) |
| --- | --- | --- | --- |
| Animal husbandry |  |  |  |
| Feed management |  |  |  |
| Animal health management |  |  |  |
| Other (Specify____) |  |  |  |
| Other (Specify____) |  |  |  |
| Other (Specify____) |  |  |  |
| Other (Specify____) |  |  |  |

81. What areas of training do you think will improve your animal health and productivity?

……………………………………………………………………………………………………………………………………………………………………………………………………

Thank you for your time and Cooperation
