# Supplementary material for: Small ruminant production system characteristics and their influence on use, alternatives of, and resistance to antimicrobials in Nigeria
Source: BMC Vet Res. 2026 Jun 20;22:602. doi: 10.1186/s12917-026-05577-7 (PMC13663043; doi:10.1186/s12917-026-05577-7)
Supplement: Supplementary file 2 [file 12917_2026_5577_MOESM3_ESM.docx]

**Supplementary Table 1- Flock distribution and size**

| **Species** | **Gender** | **Age** | **Median (Range)** |
| --- | --- | --- | --- |
| Goat | Male | < 6 months | 2 (0 - 58) |
|  |  | 6 months – 2 years | 2 (0 - 30) |
|  |  | >2 years | 2 (0 - 60) |
|  | Female | < 6 months | 2 (0 - 25) |
|  |  | 6 months – 2 years | 2 (0 - 22) |
|  |  | >2 years | 3 (0 - 30) |
| Sheep | Male | < 6 months | 2 (0 - 60) |
|  |  | 6 months – 2 years | 2 (0 - 45) |
|  |  | >2 years | 2 (0 - 60) |
|  | Female | < 6 months | 2 (0 - 32) |
|  |  | 6 months – 2 years | 3 (0 - 43) |
|  |  | >2 years | 4 (0 - 100) |
