# Supplementary material for: Small ruminant production system characteristics and their influence on use, alternatives of, and resistance to antimicrobials in Nigeria
Source: BMC Vet Res. 2026 Jun 20;22:602. doi: 10.1186/s12917-026-05577-7 (PMC13663043; doi:10.1186/s12917-026-05577-7)
Supplement: Supplementary file 3 [file 12917_2026_5577_MOESM4_ESM.docx]

**Supplementary Table 2: Results from bivariate analysis.**

| **Variables** | **Categories** | **Knowledge** | | | | **Perceptions** | | | | **Practices** | | | | |
| --- | --- | --- | --- | --- | --- | --- | --- | --- | --- | --- | --- | --- | --- | --- |
|  |  | **Satisfactory**  **n (%)** | **Unsatisfactory n (%)** | **χ² (df)** | **p-value** | **Satisfactory n (%)** | **Unsatisfactory n (%)** | **χ² (df)** | **p-value** | **Satisfactory**  **n (%)** | **Unsatisfactory n (%)** | **χ² (df)** | **p-value** |  |
| **Age** | <20 years | 1 (12.50) | 7 (87.50) | 9.38 (1) | 0.095 | 5 (62.50) | 3 (37.50) | 25.38 (5) | **<0.001** | 7 (87.50) | 1 (12.50) | 6.75 (5) | 0.239 |  |
|  | 21 – 30 years | 57 (43.85) | 73 (56.15) |  |  | 86 (66.15) | 44 (33.85) |  |  | 71 (54.62) | 59 (45.38) |  |  |  |
|  | 31 – 40 years | 92 (33.21) | 185 (66.79) |  |  | 156 (56.31) | 121 (43.69) |  |  | 153 (55.23) | 124 (44.77) |  |  |  |
|  | 41 – 50 years | 83 (41.50) | 117 (58.50) |  |  | 102 (51.00) | 98 (49.00) |  |  | 114 (57.00) | 86 (43.00) |  |  |  |
|  | 51 – 60 years | 48 (36.36) | 84 (63.64) |  |  | 59 (44.69) | 73 (55.31) |  |  | 75 (56.82) | 57 (43.18) |  |  |  |
|  | >60 years | 18 (47.37) | 20 (52.63) |  |  | 10 (26.31) | 28 (73.69) |  |  | 27 (71.05) | 11 (28.95) |  |  |  |
| **Gender** | Male | 225 (36.70) | 388 (63.29) | 1.64 (1) | 0.200 | 300 (48.94) | 313 (51.06) | 20.08 (1) | **<0.001** | 361 (58.89) | 252 (41.11) | 3.98 (1) | **0.046** |  |
|  | Female | 74 (43.02) | 98 (56.98) |  |  | 118 (68.60) | 54 (31.40) |  |  | 86 (50.00) | 86 (50.00) |  |  |  |
| **State** | Kwara | 89 (23.60) | 288 (76.39) | 63.33 (1) | **<0.001** | 106 (28.11) | 271 (71.89) | 182.09 (1) | **<0.001** | 269 (71.35) | 108 (28.65) | 60.31 (1) | **<0.001** |  |
|  | Niger | 210 (51.47) | 198 (48.53) |  |  | 312 (76.47) | 96 (23.53) |  |  | 178 (43.63) | 230 (56.27) |  |  |  |
| **Level of**  **Education** | No formal education | 127 (27.55) | 334 (72.45) | 61.63 (3) | **<0.001** | 184 (39.91) | 277 (60.09) | 80.81 (3) | **<0.001** | 276 (56.87) | 185 (40.13) | 9.63 (3) | **0.022** |  |
|  | Primary School | 48 (42.11) | 66 (57.89) |  |  | 78 (68.42) | 36 (31.58) |  |  | 69 (60.53) | 45 (39.47) |  |  |  |
|  | Secondary School | 76 (59.38) | 52 (40.63) |  |  | 95 (74.22) | 33 (25.78) |  |  | 58 (45.31) | 70 (54.69) |  |  |  |
|  | Tertiary Education | 48 (58.54) | 34 (41.46) |  |  | 61 (74.39) | 21 (25.61) |  |  | 44 (53.66) | 38 (46.34) |  |  |  |
| **Role on**  **Farm** | Owner | 163 (35.36) | 298 (64.64) | 23.32 (3) | **<0.001** | 251 (54.45) | 210 (45.55) | 15.64 (3) | **0.001** | 280 (60.74) | 181 (39.26) | 14.02 (3) | **0.002** |  |
|  | Owner and manager | 89 (35.89) | 159 (64.11) |  |  | 115 (46.37) | 133 (53.63) |  |  | 124 (50.00) | 124 (50.00) |  |  |  |
|  | Manager | 28 (54.90) | 23 (45.10) |  |  | 31 (60.78) | 20 (39.22) |  |  | 34 (66.67) | 17 (33.33) |  |  |  |
|  | Worker | 19 (76.00) | 6 (24.00) |  |  | 21 (84.00) | 4 (16.00) |  |  | 9 (36.00) | 16 (64.00) |  |  |  |
| **Number of years in ruminant farming** | ≤10 years | 119 (44.24) | 150 (55.76) | 11.54 (4) | 0.021 | 198 (73.61) | 71 (26.40) | 89.08 (4) | **<0.001** | 128 (47.58) | 141 (52.42) | 17.66 (4) | **0.001** |  |
|  | 11-20 years | 102 (39.53) | 156 (60.47) |  |  | 132 (51.16) | 126 (48.84) |  |  | 156 (60.47) | 102 (39.53) |  |  |  |
|  | 21-30 years | 43 (29.66) | 102 (70.34) |  |  | 42 (28.97) | 103 (71.03) |  |  | 98 (67.59) | 47 (32.41) |  |  |  |
|  | 31 – 40 years | 24 (32.43) | 50 (67.57) |  |  | 34 (45.95) | 40 (54.06) |  |  | 43 (58.11) | 31 (41.89) |  |  |  |
|  | >40 years | 11 (28.20) | 28 (71.80) |  |  | 12 (30.77) | 27 (69.23) |  |  | 22 (56.41) | 17 (43.59) |  |  |  |
| **Herd size of**  **animals** | ≤25 | 110 (36.79) | 189 (63.21) | 2.11 (3) | 0.550 | 170 (56.86) | 129 (43.14) | 2.69 (3) | 0.442 | 179 (59.87) | 120 (40.13) | 6.07 (3) | 0.108 |  |
|  | 26-50 | 135 (39.02%) | 211 (60.98) |  |  | 175 (50.58) | 171 (49.42) |  |  | 185 (53.47) | 161 (46.53) |  |  |  |
|  | 51-75 | 35 (43.21%) | 46 (56.79) |  |  | 43 (53.09) | 38 (46.91) |  |  | 43 (53.09) | 38 (46.91) |  |  |  |
|  | >75 | 19 (32.20%) | 40 (67.80) |  |  | 30 (50.85) | 29 (49.15) |  |  | 40 (67.79) | 19 (32.20) |  |  |  |
