# Supplementary material for: Small ruminant production system characteristics and their influence on use, alternatives of, and resistance to antimicrobials in Nigeria
Source: BMC Vet Res. 2026 Jun 20;22:602. doi: 10.1186/s12917-026-05577-7 (PMC13663043; doi:10.1186/s12917-026-05577-7)
Supplement: Supplementary file 4 [file 12917_2026_5577_MOESM5_ESM.docx]

**Additional File 2 - Interview Guide**

1. WHAT ARE THE MAIN CHALLENGES THAT AFFECT YOUR HERD?
i. What are the most important ones, and in what order?
ii. How much do they cost you in milk and meat production, economic losses, etc.?
2. WHAT DO YOU OR OTHER FARMERS DO WHEN THEY HAVE THESE DISEASES?
i. Who do they consult? Can you rank these in order of important ones (veterinarians, para veterinarians)?
ii. What do they use (drugs, nothing, etc.)? Can you rank these in order of importance?
iii. Why do they consult who they do? Why do they use what they use?
3. DO YOU RELY ON YOUR EXPERIENCE, OR DO YOU DO CONSULTATION, WHEN YOU HAVE THESE ISSUES?
i. What makes you decide to rely on your own experience or consult people?
4. WHAT DOES MASTITIS MEAN TO YOU?
i. How do you know when you have mastitis?
ii. Do you take samples to the lab?
iii. Do you call a veterinarian?
iv. If not, why?
5. HOW MUCH DOES MASTITIS AFFECT YOUR HERD EVERY YEAR?
i. How much have you lost from sales due to mastitis?
ii. How much have you lost from milk production?
iii. How much was the treatment cost?
6. WHAT DO YOU USE IN PREVENTING MASTITIS?
i. How do you prevent it so it doesn’t happen to you?
ii. Rank these in order of importance.
7. WHAT DO YOU USE IN TREATING MASTITIS?
i. How do you cure it so you don’t lose as much?
ii. Rank these in order of importance.
8. HOW DO FARMERS IN YOUR AREA PREVENT AND TREAT MASTITIS?
i. What do they do to prevent and treat mastitis?
ii. Rank these methods in order of how well they work?
iii. Which methods are preferred, and why?
9. WHO DO YOU CONSULT WHEN YOU HAVE MASTITIS?
i. Veterinarians, Para-veterinarians, no one?
ii. Rank these consultants in order of how well they work?
iii. If you don’t use vets, why?
10. DO YOU USE ANTIBIOTICS?
i. Where do you get them from?
ii. What drugs do you use?
iii. Rank them in order of importance.
11. DO YOU USE ALTERNATIVES TO ANTIBIOTICS?
i. Where do you get them from?
ii. What do you use?
iii. Do they work as well as antibiotics?
iv. Do you have any concerns with using alternatives to antibiotics rather than antibiotics?
